# Real-time prediction of cardiorespiratory deterioration during paediatric critical care transport using interpretable machine learning

**DOI:** 10.1371/journal.pdig.0001410

**Published:** 2026-05-19

**Authors:** Milan Kapur, Kezhi Li, Alexander Brown, Zhiqiang Huo, John Booth, Philip Knight, Gwyneth Davies, Padmanabhan Ramnarayan

**Affiliations:** 1 Department of Population, Policy and Practice, UCL Great Ormond Street Institute of Child Health, London, United Kingdom; 2 Institute of Health Informatics, University College London, Euston Road, London, United Kingdom; 3 Department of Infection, Immunity and Inflammation, UCL Great Ormond Street Institute of Child Health, London, United Kingdom; 4 Wolfson Institute of Population Health, Queen Mary University of London, London, United Kingdom; 5 Department of Population Health Sciences, King’s College London, London, United Kingdom; 6 Digital Research Innovation and Virtual Environment (DRIVE), Great Ormond Street Hospital, London, United Kingdom; 7 Children’s Acute Transport Service (CATS), Great Ormond Street Hospital for Children NHS Foundation Trust, London, United Kingdom; 8 Department of Respiratory Medicine, Great Ormond Street Hospital for Children NHS Foundation Trust, London, United Kingdom; 9 Department of Surgery and Cancer, Imperial College London, London, United Kingdom; 10 Department of Paediatric Critical Care, St Mary’s Hospital, Imperial College NHS Healthcare Trust, London, United Kingdom; Southeast University, CHINA

## Abstract

Interhospital transport of critically ill children carries inherent risks, including unexpected respiratory and cardiovascular deterioration. Early warning of impending patient deterioration may allow physicians to intervene and prevent further decline. We developed and evaluated lightweight, explainable machine learning models to forecast adverse physiological events up to 15 minutes in advance using continuously streamed vital signs and clinical data. Models were trained and evaluated on 1,519 transports conducted by a specialist paediatric critical care team in London (2016–2021). Transformer-based models incorporating vital sign time-series and vector-embedded diagnoses outperformed simpler models, achieving AUROC scores of 0.851 for respiratory and 0.792 for cardiovascular deterioration. Model interpretability was provided using Integrated Gradients, revealing alignment with clinical reasoning. Designed for deployment on edge devices, these models offer real-time, interpretable risk predictions in resource-limited transport settings. These results demonstrate that real-time, explainable machine learning models can accurately predict deterioration during interhospital paediatric transport using routinely collected data, supporting their potential role in enhancing early clinical intervention.

## Author summary

We developed two lightweight, explainable artificial intelligence (AI) models to predict critical respiratory and cardiovascular complications in seriously ill children during hospital-to-hospital transfer. Transporting these children is a high-risk process, with a chance of unexpected, dangerous changes in their condition. Currently, transport teams rely on manually interpreting vital signs, which can be challenging because “normal” ranges for critically unwell children vary widely.

Our AI models use the patient’s continuous, high-frequency vital signs, like heart rate and blood pressure, combined with clinical information such as their primary diagnosis. We trained them on data from over 1,500 transport episodes conducted by a specialist team in London. Crucially, the models are designed to be run in real-time on portable devices, providing a prediction of deterioration up to 15 minutes in advance.

A key strength of our work is that the models are explainable, meaning they tell clinicians *why* they made a prediction, with the reasons often aligning with clinical reasoning. By providing this early warning and interpretability, the AI has the potential to help doctors and nurses intervene sooner, enhancing patient safety and potentially improving outcomes in a resource-limited setting.

## Introduction

In the United Kingdom, around 20,000 children require admission to Paediatric Intensive Care Units (PICUs) annually [1]. Many initially present to general hospitals that lack on-site PICU services, making urgent interhospital transfer essential for them to receive necessary intensive care. In 2023, more than 4,000 such transfers were conducted [1]. Despite being conducted by specialised Paediatric Critical Care Transport (PCCT) teams of doctors, nurses, and paramedics, interhospital transfers remain high-risk [2]. Transport-related physiological stress elevates the risk of en-route clinical deterioration, with adverse events reported in 12.3% of over 8,000 interhospital transport episodes in one large study [3]. A recent systematic review categorised transport associated adverse events into four main groups: respiratory (e.g., hypoxaemia), cardiovascular (e.g., hypotension, tachycardia/bradycardia), equipment-related (e.g., monitor failure), and other (e.g., medication error) [4]. These events, even when promptly identified and managed by PCCT teams, can lead to significant morbidity and even mortality, underscoring the critical need for advancements that move beyond reactive care to proactive intervention.

During transport, vital signs are continuously monitored to detect deterioration. However, PCCT services largely rely on manual interpretation against static, age-based reference ranges or clinical judgment. This approach has limitations, as “normal” vital sign ranges for critically unwell children vary widely with age, diagnosis, and illness severity. However, this approach requires well-characterized normal vital sign ranges for the patient. Whilst these ranges exist for stable paediatric patients, there are no “normal” ranges for critically unwell children during transport; the expected values vary significantly depending on age, diagnosis, severity of illness and ongoing interventions [5–8]. This often leads to either over-alerting (false alarms due to transient fluctuations in already abnormal physiology) or under-detection of subtle but significant changes. To address this, our prior work explored dynamic thresholding approaches that better account for inter-individual variability, allowing for personalised and adaptive detection of patient deterioration [9].

While detecting deterioration in real time is critical for timely intervention, predicting future deterioration enables clinicians to act pre-emptively before a patient’s condition worsens. Recent machine learning (ML) advancements allow sophisticated predictive tools to leverage large patient datasets, analysing continuous physiological data to forecast acute deterioration [10–14]. Despite these advancements, to the best of our knowledge, no models exist capable of predicting real-time risk of acute deterioration during paediatric critical care transport. To address this gap, we introduce two novel, lightweight ML models capable of predicting respiratory and cardiovascular deterioration up to 15 minutes in advance, in real-time. Trained on a large dataset of 1200 transport episodes with a lightweight design suitable for edge computation, these models have the potential to provide timely clinical decision support during transport, which may in turn enable earlier intervention and contribute to improved patient outcomes.

## Methods

### Study approval

The study was approved by Great Ormond Street Hospital’s (GOSH) Research and Innovation Department, under ethical approvals for use of routine de-identified healthcare and operational hospital data (Research Database, NHS REC reference 21/LO/0646).

### Data sources

**Fig 1:**
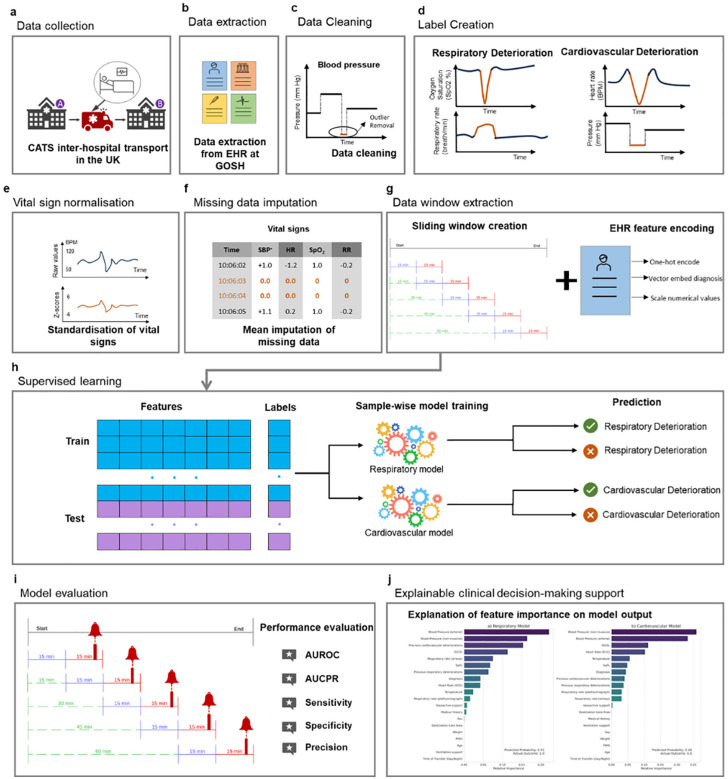
a) Vital sign data were collected during the interhospital transfer. **b)** Corresponding demographic and clinical information were extracted from the EHR at GOSH. **c)** Data was cleaned with physiologically implausible values removed. **d)** Respiratory and cardiovascular events were labelled using a Bollinger Bands-based method, applying patient-specific dynamic thresholds to identify sustained combinations of significant deviations in vital signs. **e)** Vital signs were normalised/standardised to allow efficient training. **f)** Missing vital sign values were mean imputed. **g)** 15-minute windows of data were extracted to convert each transport episode into multiple windows of data. These windows were combined with the demographic and clinical features derived from the EHR to make a complete feature set for the window. Care was taken to ensure data from a given transport episode belonged exclusively to either the train, tune or test sets. **h)** Models were then trained for the classification task: to predict whether an adverse respiratory or cardiovascular event occurred in the 15-minute label period. **i)** Model performance was evaluated using a range of metrics. **j)** Integrated Gradients were used to understand the features that drive the model to make predictions at a given time-point at a cohort and individual level.

This retrospective study analysed continuously monitored vital signs of critically ill children transported by the Children’s Acute Transport Service (CATS), a regional paediatric critical care team in North London, UK, between July 2016 and May 2021. Since 2016, CATS has used SwiftCare (Kinseed Limited, UK) to collect one data point per second vital signs, including heart rate, respiratory rate, blood pressure, oxygen saturation (SpO2), and end-tidal carbon dioxide (EtCO2). Data collection starts at transfer initiation, with ambulance staff using a SwiftCare-enabled smartphone to connect wirelessly to a Philips Intellivue MP5 monitor, recording continuously until patient handover at the destination unit (Fig 1). To calculate the total transport duration for each episode, we utilized the UTC timestamps associated with the high-frequency vital sign data. Transport time was defined as the temporal difference between the timestamp of the first recorded data point (corresponding to monitor connection at transfer initiation) and the last recorded data point (corresponding to disconnection at patient handover). Data capture during the study period was limited by operational constraints; specifically, only two recording devices were available across the three active transport teams, making full cohort coverage unfeasible. Additional data loss occurred on occasions when devices were inadvertently not deployed or experienced technical connectivity issues. These data were linked with de-identified electronic health records (EHRs), which included demographics, transport details, diagnoses, pre-transport interventions, intra-transport support, and time-series vital signs. A complete list of recorded parameters is provided in Table 1. Not all parameters were recorded for every patient due to clinical variability (e.g., EtCO₂ was typically only recorded for invasively ventilated patients). While the majority of transport episodes involved unique patients, a small minority may reflect repeat transports. Due to record anonymisation, we could not identify repeated episodes for the same patient. However, as each transport was clinically distinct, all episodes were treated as independent records.

**Table 1:** List of collected Electronic Health Care (EHR) and vital sign data in the cohort.

Category	EHR and vital signs data	Data type	Range	Missing (%)
Patient demographics	Age (years),	Numerical	0-18	0.00%
	Weight (kg)	Numerical	1-90	0.26%
	Sex	Categorical	NA	0.07%
	Ethnicity	Categorical	NA	51.68%
Transport details	Referring hospital	Categorical	NA	0.00%
	Destination hospital	Categorical	NA	0.26%
	Time of transfer	Numerical	NA	0.00%
	Destination care area (PICU, NICU, HDU Ward, other)	Categorical	NA	0.20%
Medical Diagnosis	Primary diagnosis,	Categorical	NA	0.00%
	Paediatric index of mortality 3 (PIM3),	Numerical	0-1	0.26%
	Existing medical conditions (respiratory, cardiac, renal, genetic, metabolic/endocrine, haematological/oncological, other)	Categorical	NA	0.00%
Interventions by local team prior to transport	Intubation (primary/re-intubation/ETT repositioning), mechanical ventilation (invasive, non‐invasive and HFNC), suctioning, chest drain insertion, vascular access (peripheral, central, arterial, intraosseous), vasoactive support (inotropes/vasopressors, prostaglandin), blood product transfusion, urinary catheterisation, nasogastric/orogastric tube placement, imaging (CT scan), C-spine immobilisation, osmotherapy, CPR/defibrillation, ECMO, and ICP monitoring	Binary	NA	0.79%
Intra-transport respiratory support commenced prior to transport	Self-ventilating: Room air, Supplemental O₂, HFNC, CPAP, BiPAP; Invasive ventilation: ETT, Tracheostomy, Other airway	Categorical	NA	0.72%
Intra-transport cardiovascular support commenced prior to transport	Adrenaline, noradrenaline, dobutamine, dopamine, milrinone, prostaglandin, inhaled nitric oxide	Categorical	NA	0.00%
Vital Signs	SpO2	Numerical	0-100	0.07%
	Heart rate (3-lead ECG)	Numerical	0-301	0.59%
	End tidal CO2 (minimum value in 1-second period)	Numerical	0-19.1	29.00%
	End tidal CO2 (maximum value in 1-second period)	Numerical	0.3-20.1	27.32%
	Airway derived respiratory rate	Numerical	0-164	27.39%
	Impedance pneumography derived respiratory rate	Numerical	0-171	4.08%
	Mean systolic blood pressure (non-invasive)	Numerical	0-264	5.99%
	Mean arterial blood pressure (non-invasive)	Numerical	0-242	5.99%
	Mean diastolic blood pressure (non-invasive)	Numerical	0-247	5.99%
	Mean systolic blood pressure (invasive)	Numerical	0-361	63.98%
	Mean arterial blood pressure (invasive)	Numerical	0-361	63.98%
	Mean diastolic blood pressure (invasive)	Numerical	0-340	63.98%
	Temperature (oesophageal)	Numerical	2.9-40.8	78.74%
	Temperature (skin)	Numerical	6.6-43.4	72.55%
	Temperature (core)	Numerical	8.2-44.6	85.71%
	Temperature (unspecified)	Numerical	5.8-43.1	64.05%

Missing rates were calculated on the 1519 patients included in the study and captures transport episodes where there were zero recorded values of a given parameter. PICU = Paediatric Intensive Care Unit, NICU = Neonatal Intensive Care Unit, HDU = High Dependency Unit, ETT = Endotracheal tube, HFNC = High Flow Nasal Canula, CPR = Cardiopulmonary Resuscitation, ECMO = Extra-Corporeal Membrane Oxygenation, ICP = Intracranial Pressure, CPAP = Continuous Positive Airway Pressure, BiPAP = Bi-level Positive Airway Pressure.

### Inclusion criteria

Episodes were included if the patient was ≤ 18 years old with at least 30 minutes of recorded vital signs. This 30 minute threshold was selected to ensure a minimum of 15 minutes of high-resolution input data to establish a physiological baseline, followed by at least one 15-minute window for prediction and labelling. Given the nature of inter-hospital specialist transport, which includes stabilization and handover phases, 30 minutes represents a minimal clinical duration; thus, this criterion was not expected to exclude high-acuity “scoop and run” cases, but rather to ensure the models had sufficient temporal context for meaningful inference. Continuous data throughout transport was not mandated, acknowledging real-world interruptions such as sensor dropouts or new measurements (e.g., EtCO2 upon intubation during transport).

### Data processing

Vital sign data, for most parameters was collected at one-second resolution. For parameters with lower sampling frequencies (temperature and Non-Invasive Blood Pressure), a ‘last observation carried forward’ (LOCF) approach was used between measurements. Physiologically implausible values were removed from the vital sign data (heart rate <30 or >300 bpm, SpO2 < 10 or >100%, EtCO₂ > 15 kPa, blood pressure <5 mmHg, temperature <25°C or >45°C). To account for age-related variability, all vital signs were standardised to improve comparability and enhance the reliability of model training. Respiratory rate, heart rate, and blood pressure were z-score normalised following methodology outlined in our prior work [5]. SpO₂ was scaled using (SpO₂ − 97)/6, ETCO₂ using (ETCO₂ − 5.25)/1.5, and temperature using (Temp − 36.75)/1.5.

Missing values (data due real-world interruptions such as sensor dropouts) were mean-imputed (set to zero post-standardisation). Beyond random sensor dropouts, sustained missingness was treated as ‘informative missingness’ to capture clinical intent. For example, the high missingness rate for Invasive Blood Pressure (63.98%) reflects the proportion of patients without arterial access; preserving this allowed the model to learn the implicit clinical assessment of cardiovascular stability associated with the decision not to insert an arterial line.To enable the model to distinguish between observed and imputed data, a binary missingness mask (1 = observed, 0 = imputed) was generated for each of the 18 input features. This mask was concatenated with the physiological values along the feature dimension, resulting in a dual-channel input vector of 36 features per time step.

Demographic features were pre-processed for model compatibility. Age, weight, and the Paediatric Index of Mortality 3 (PIM3) were scaled to a 0–1 range, with missing values imputed as –1 [15]. Sex was binary encoded, and ethnicity one-hot encoded using NHS Digital schema [16]. Referring and destination hospitals were also one-hot encoded, with units having <10 retrievals grouped as “Other.” One-hot encoding was applied to destination care area, pre-transport interventions, intra-transport respiratory and cardiovascular support, and pre-existing conditions. Team arrival time was used to label day vs night shifts. The EHR included the primary diagnosis (the acute reason for the critical care transport) and existing medical conditions (documented chronic co-morbidities such as respiratory, cardiac, or neurological baseline conditions). Primary diagnosis was encoded in two ways: via one-hot encoding into clinical categories (respiratory, cardiovascular, neurological, infection, gastroenterology, metabolic, trauma), and via 768-dimensional embeddings from BioClinicalBERT, pre-trained on MIMIC-III [17]. Embeddings were precomputed and cached in advance to avoid inference-time overhead. Both encoding strategies were tested during model development. Existing co-morbidities were already grouped by organ system and were one-hot encoded. To evaluate the minimal configuration required for real-time deployment, we compared two baseline feature sets. The ‘Full Baseline’ configuration utilized 30 variables from the EHR, including all demographic data and the exhaustive list of pre-transport clinical interventions shown in Table 1. The ‘Reduced Baseline’ configuration was restricted to 9 core clinical variables: age, weight, sex, PIM3 score, destination care area, primary diagnosis, pre-existing medical conditions, and intra-transport respiratory and cardiovascular support. This reduction intentionally excluded sparse or inconsistently recorded demographic fields and was intended to minimise the manual data entry burden for clinicians during high-acuity transport, ensuring that the model requires only the nine most critical clinical variables for immediate inference.

### Label creation

Our dataset did not include timestamped, clinician-annotated deterioration labels. To address this, we implemented an automated, data-driven approach adapting Bollinger bands to continuous vital sign data, as detailed in our previous work [18]. For each transport episode, one-minute mean-averaged data were used to compute an exponentially weighted moving average (EMA) and standard deviation (EWMSTD), generating patient-specific upper and lower thresholds that reflect each individual’s evolving baseline. To prevent these dynamic bands from becoming infinitesimally narrow during periods of prolonged stability or low variability if a parameter’s EWMSTD was within 5% of its current EMA, a fixed threshold of ±5% of the current EMA was enforced. For oxygen saturation (SpO₂), due to its inherently lower variability, a stricter fixed boundary of ±2.5% of its current EMA was applied. Respiratory deteriorations were flagged when oxygen saturation (SpO₂) fell below either a fixed threshold of 94% or the dynamic lower bound (whichever was lower) alongside a concurrent abnormality in at least one other respiratory parameter (impedance pneumography derived respiratory rate, airway-derived respiratory rate, or EtCO₂). Cardiovascular deteriorations were identified by simultaneous deviations in heart rate and blood pressure. To reduce false positives from transient fluctuations or artefacts, events were required to persist for at least one minute and be flanked by five minutes of continuous data. These criteria were applied retrospectively to label minute-level or cumulative periods of respiratory and cardiovascular deterioration.

Crucially, our automated labelling method does not carry the inherent risk of a model “rediscovering” the labelling logic. This is because the model’s prediction relies exclusively on preceding 15-minute vital sign data and historical context, not on the vital sign data within the 15-minute prediction window itself. This strict temporal separation ensures that the model cannot simply identify the conditions that trigger a band-based alert at the time of the event. Instead, the model is designed to learn and predict future deterioration based on subtle physiological shifts and patterns that precede a deterioration event, rather than merely re-identifying the criteria used to define the event retrospectively.

### Data split

We applied a label-stratified split, allocating 80% of episodes (1,214) to training, 10% (150) to tuning, and 10% (155) to testing. The slight imbalance between tuning and test set sizes reflects the stratification process. Episodes were first grouped into four categories based on deterioration patterns: no deterioration, only respiratory deterioration, only cardiovascular deterioration, and both respiratory and cardiovascular deterioration. Each category was then split into 80/10/10 subsets, which were subsequently combined to form the final stratified dataset.

### Window creation

**Fig 2:**
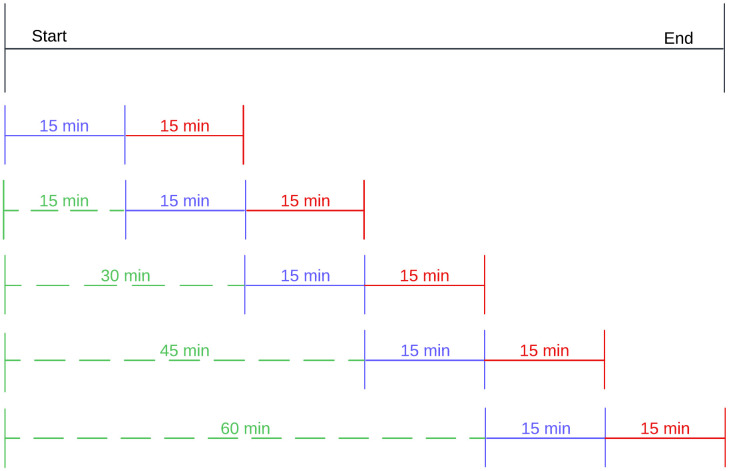
This diagram illustrates how each transport episode is divided into sequential time windows for predictive modelling of adverse events. The 15-minute interval highlighted in red is the prediction window, during which adverse events may occur. The 15-minute interval in blue represents the immediately preceding 15-minute period where vital signs are captured at 1-second resolution. The green section indicates an extended historical context (up to a maximum of 120 minutes prior), where vital signs are down-sampled to 5-minute averages to reduce computational overhead while preserving key trends. Each horizontal “track” corresponds to a different 15-minute prediction window, demonstrating how multiple windows are formed across the full duration of a transport episode.

To facilitate model training and evaluation time series vital signs data was first discretised into 15-minute windows. This ensured that each period was used only once as a potential label window. For each window the model was tasked to use the data from the current window (*t*_-14:59_ - *t*_00:00_), to predict whether any adverse event would occur in the next window (*t*_00:01_ - *t*_+15:00_). Timestamped adverse events from the current window were also included as features. If data was available prior the current window (as would occur with data accumulation over the course of a long transport episode) additional context was provided as input to the model by summarizing data from *t*_-02:15:00_ up to *t*_-00:15:00_. This additional data was mean-averaged over 5-minute intervals and each interval was annotated with the occurrence of respiratory or cardiovascular events, further enriching the feature set. Strict controls were applied to prevent future information leakage, and all windows from a given episode remained within the same data split (train, tune, or test). Fig 2 illustrates this windowing strategy.

We chose a 15-minute prediction horizon and a 15-minute high-resolution input window due to pragmatic and clinical considerations. This future horizon offers clinically relevant lead time for proactive intervention without exceeding the model’s predictive capability or providing insights that are not sufficiently actionable. The 15-minute input window effectively establishes a robust clinical baseline and captures meaningful physiological trends.12 Shorter windows (e.g., 10 minutes) might lack sufficient context, while longer ones (e.g., 20 minutes) would increase computational complexity and latency, hindering real-time deployment without substantial predictive gains. This duration also supports frequent predictions during typical transport times (median 113 minutes). Additionally, 120 minutes of averaged historical context (at 5-minute intervals) was included to offer a broader clinical trajectory while maintaining computational efficiency for edge device deployment.

### Model development

We developed two sets of models to forecast respiratory and cardiovascular deteriorations within a 15-minute horizon. Six models were developed for each prediction task, progressively increasing in model complexity and feature richness to identify the minimal configuration needed to sustain high performance while minimising user input and computational load (see S1 Table). We used a transformer architecture for time-series analysis of vital signs, leveraging its superior ability to model long-range dependencies and its computational efficiency over recurrent approaches such as recurrent neural networks or long short-term memory networks [19]. To optimize temporal encoding, transformer blocks were implemented with rotary positional embeddings [20]. Transformer blocks were implemented with a decoder-only architecture to ensure data points could only attend to preceding data, thereby guaranteeing that predictions were based solely on historical information [19]. Given the class imbalance (adverse events being rare) we applied class-weighted loss during training to penalize false negatives more heavily to maintain balanced performance across classes.

We performed random search hyper-parameter optimization over the training and tuning sets exploring parameters including learning rate, batch size, number of epochs, positional weighting, hidden layer dimensions, transformer heads and layers, batch normalization, dropout rates, L2 regularization, and max-norm constraints to identify the optimal training configuration. The best-performing model on the tuning set was then evaluated on the test set. This approach yielded separate models for predicting respiratory and cardiovascular events. While a combined multiclass model was considered, the non-exclusive nature of events and differing optimal architectures (e.g., hidden layer sizes, transformer depth, attention heads) made separate models more practical.

### Model evaluation

Model performance was evaluated on the label-stratified holdout test set. We calculated the Area Under the Receiver Operating Characteristic Curve (AUROC) and the Area Under the Precision-Recall Curve (AUPRC) to assess discrimination performance across all possible thresholds. In addition, we calculated threshold-dependent metrics including sensitivity (recall), specificity, positive predictive value (PPV/precision), negative predictive value (NPV), balanced accuracy, and F1-score. To provide a robust estimate of uncertainty and ensure the reliability of our findings, 95% confidence intervals (CIs) for all performance metrics were calculated using a stratified bootstrapping approach on the test set with 5,000 iterations.

To reflect real-world clinical constraints, specifically the need to minimize alarm fatigue in a transport environment, we selected a fixed decision threshold based on the tuning set. We chose the threshold that capped the false positive rate at 20% (ensuring ≥80% specificity). This threshold was determined solely on the tuning set and applied unchanged to the test set for final evaluation. It is acknowledged that threshold-dependent metrics may vary if alternative operating points (e.g., enforcing high sensitivity) are selected.

### Model explainability

To understand the decision-making process of our models, we employed Integrated Gradients [21]. We focused on the absolute (magnitude) of each feature’s attribution, rather than its signed contribution. Because time-series features can fluctuate, sometimes pushing the prediction higher and sometimes lower, summing signed attributions could lead to misleading cancellations. By taking the absolute value, we preserved each feature’s overall contribution across time and avoided under-representing features whose positive and negative influences might otherwise cancel out. We restricted this analysis to the best-performing model configuration (Combined Transformer with Vector Diagnosis and Reduced Baseline) for both respiratory and cardiovascular tasks, selected based on AUROC performance. We applied this analysis at both the cohort level, to identify which features mattered most on average, and the individual level, to interpret specific predictions in real-time. Furthermore, to validate the clinical responsiveness of the model, we performed a temporal correlation analysis between dynamic attribution scores and raw physiological fluctuations. By mapping the Integrated Gradients attribution magnitudes against high-frequency vital sign data (1 Hz), we assessed specifically, whether spikes in risk attribution correlated with significant changes in physiological parameters. This granular time-series attribution allows for the verification of the model’s internal logic, ensuring that predictions are driven by acute physiological shifts.

### Research environment

All experiments were conducted within a secure Digital Research Environment provided by Aridhia Informatics Ltd in Glasgow, Scotland. The computational resources included an Intel Xeon Platinum 8272CL CPU with 64 GB of RAM; no GPU was utilized. Model development was performed using Python 3.12, leveraging the PyTorch framework (v2.6). Additional utilised libraries included Pandas, NumPy, X-transformers, Scikit-learn, Captum, Matplotlib, Seaborn, BioClinicalBert. We restricted this analysis to the best-performing model configuration (Combined Transformer with Vector Diagnosis and Reduced Baseline) for both respiratory and cardiovascular tasks, selected based on AUROC performance. We applied this analysis at both the cohort level, to identify which features mattered most on average, and the individual level, to interpret specific predictions in real-time. Model efficiency benchmarks were conducted weith dummy data locally on a standard consumer-grade laptop (HP EliteBook, Intel Core i7, 16GB RAM) without reliance on remote server access or GPU acceleration, to simulate the hardware constraints typical of a clinical transport environment.

## Results

### Cohort description

**Fig 3:**
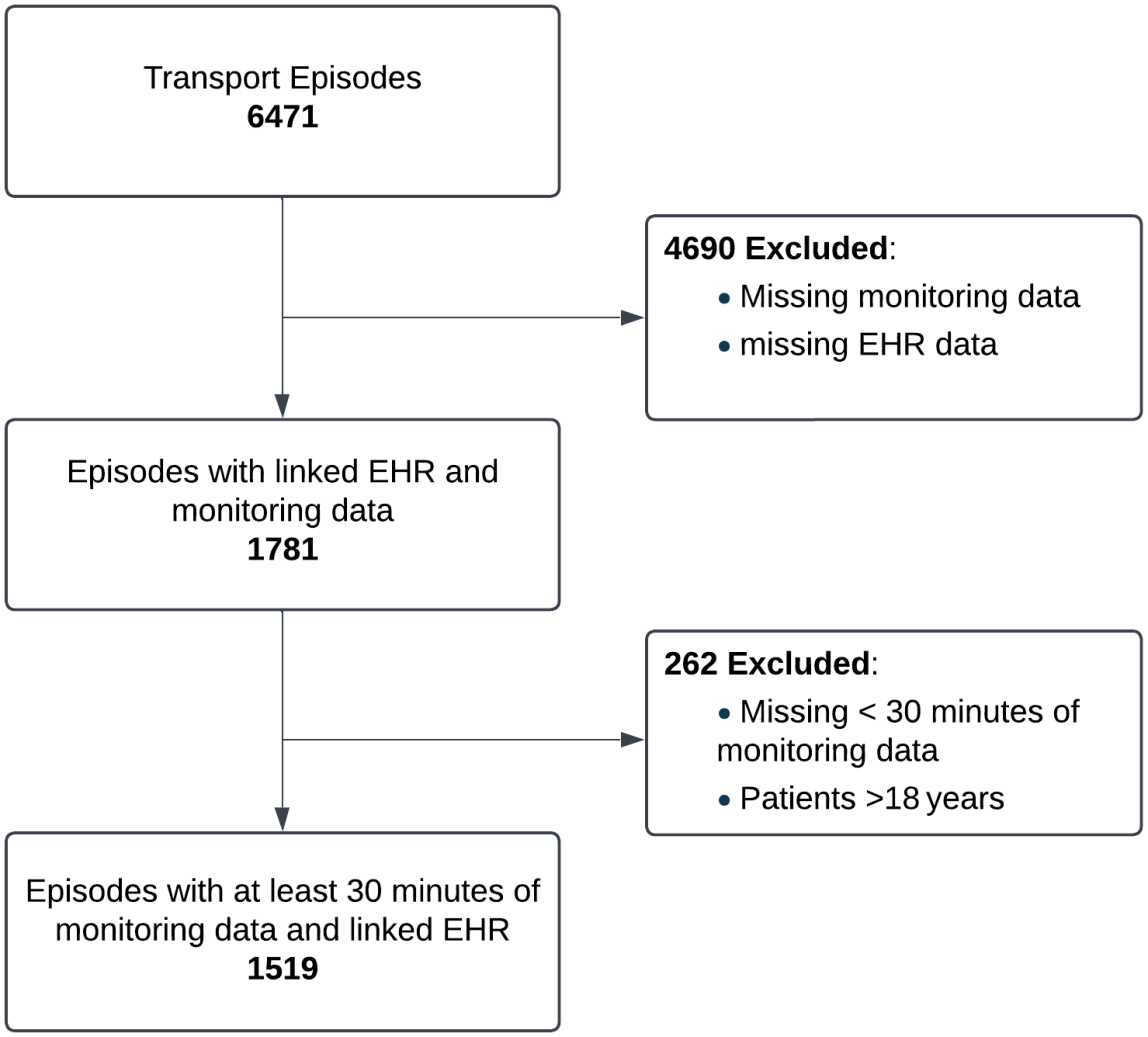
Flowchart of inclusion criteria. EHR: Electronic Health Record.

A total of 6,471 transport episodes occurred during the study period. Due to operational constraints affecting data capture, 1,781 episodes were initially available for analysis. Following the exclusion of patients aged >18 years and those with <30 minutes of monitoring data, the final cohort consisted of 1,519 episodes (23.5% of the total) (Fig 3). The included cohort was demographically and clinically representative of the overall population of transported children (Table 2). The median duration of a transport episode was 113 minutes (IQR: 75–160) (S1 Fig). The distribution of adverse events across the training, tuning, and test sets is detailed in Table 3. While approximately 31% of patients experienced at least one form of respiratory or cardiovascular deterioration during their transport, these events were rare relative to the total monitoring time. Specifically, less than 10% of all 15-minute time windows contained a deterioration event. This reflects the high level of class imbalance inherent in transport data; even patients who eventually deteriorate remain physiologically stable for the vast majority of the transfer duration. To ensure robust evaluation, all windows from an individual patient were assigned exclusively to a single data split (train, tune, or test).

**Table 2:** Breakdown of characteristics of all transport episodes and included episodes.

Characteristics	All Transport Episodes (n = 6471)	Included Episodes *(n = 1519)*
**Age Group Distribution**		
≤ 1 month	2219 (34.3%)	*568 (37.4%)*
1– ≤ 12 months	1330 (20.6%)	*296 (19.5%)*
1– ≤ 4 years	1198 (18.5%)	*264 (17.4%)*
4– ≤ 11 years	1017 (15.7%)	*239 (15.7%)*
11– ≤ 18 years	678 (10.5%)	*151 (9.9%)*
> 18 years	27 (0.4%)	*0 (0%)*
**Gender Distribution**		
Male	3603 (55.7%)	*825 (54.3%)*
Female	2861 (44.2%)	*692 (45.6%)*
**Diagnosis Group Distribution**		
Respiratory	2233 (34.5%)	*498 (32.8%)*
Cardiovascular	1385 (21.4%)	*364 (24.0%)*
Neurological	921 (14.2%)	*227 (14.9%)*
Infection	888 (13.7%)	*202 (13.3%)*
Gastrointestinal	368 (5.7%)	*89 (5.9%)*
Metabolic	161 (2.5%)	*43 (2.8%)*
Trauma	81 (1.3%)	*21 (1.4%)*
Other	434 (6.7%)	*75 (4.9%)*
**PIM3 Risk of Mortality**		
≤ 1%	502 (7.8%)	*92 (6.1%)*
1– ≤ 3%	2345 (36.2%)	*510 (33.6%)*
3– ≤ 5%	2060 (31.8%)	*518 (34.1%)*
5– ≤ 10%	1038 (16.0%)	*264 (17.4%)*
10– ≤ 15%	206 (3.2%)	*59 (3.9%)*
15– ≤ 30%	182 (2.8%)	*45 (3.0%)*
> 30%	126 (1.9%)	*30 (2.0%)*
**Respiratory Support**		
Self-ventilating (Room Air)	1292 (20.0%)	*257 (16.9%)*
Self-ventilating (supplemental O₂)	141 (2.2%)	*27 (1.8%)*
Self-ventilating (HFNC)	250 (3.9%)	*62 (4.1%)*
Self-ventilating (CPAP)	278 (4.3%)	*55 (3.6%)*
Self-ventilating (BIPAP)	54 (0.8%)	*12 (0.8%)*
Invasive ventilation (ETT)	4214 (65.1%)	*1073 (70.6%)*
Invasive ventilation (Tracheostomy)	93 (1.4%)	*19 (1.3%)*
Invasive ventilation (Other airway)	10 (0.2%)	*3 (0.2%)*
**Cardiovascular Support**		
Adrenaline	815 (12.6%)	*199 (13.1%)*
Dobutamine	31 (0.5%)	*9 (0.6%)*
Dopamine	580 (9.0%)	*137 (9.0%)*
Milrinone	58 (0.9%)	*12 (0.8%)*
Noradrenaline	507 (7.8%)	*131 (8.6%)*
Any agent	2011 (31.1%)	*488 (32.1%)*
**Overall Transport Time, minutes**		
≤ 60	16 (0.2%)	*0 (0.0%)*
60–120	598 (9.2%)	*105 (6.9%)*
120–180	1523 (23.5%)	*345 (22.7%)*
180–240	2067 (31.9%)	*545 (35.9%)*
240–300	1282 (19.8%)	*322 (21.2%)*
300–360	556 (8.6%)	*142 (9.3%)*
> 360	261 (4.0%)	*55 (3.6%)*

Demographic, clinical, and transport characteristics of the study population compared to all transported children during the study period. Respiratory support and cardiovascular support refer to support received during transport. HFNC = High-Flow Nasal Cannula; CPAP = Continuous Positive Airway Pressure; BIPAP = Bilevel Positive Airway Pressure; ETT = Endotracheal Tube.

**Table 3:** Frequency of adverse events in time windows.

Metric	Train	Tune	Test
Patient Level Analysis	n = 1214	n = 150	n = 155
Only Respiratory Deterioration (%)	9.55%	9.15%	9.30%
Only Cardiac Deterioration (%)	15.32%	15.15%	15.72%
Both Respiratory and Cardiac Deterioration (%)	6.18%	6.00%	6.45%
No Deterioration (%)	68.90%	69.70%	68.53%
			
Window-Level Analysis (15-min segments)	n = 8109	n = 1022	n = 1031
Only Respiratory Deterioration (%)	2.48%	2.25%	3.1%
Only Cardiac Deterioration (%)	4.06%	5.19%	4.36%
Both Respiratory and Cardiac Deterioration (%)	0.57%	0.59%	0.87%
No Deterioration (%)	92.9%	91.98%	91.66%

This table summarizes the total number of patients and time windows analysed in the train, tune, and test sets, along with the percentages of patients and time windows exhibiting respiratory deterioration, cardiac deterioration, both respiratory and cardiac deterioration, and no deterioration. Notably,whilst approximately 30% of patients demonstrated some form of either respiratory or cardiovascular deterioration, this translated to less that 10% of time windows. This difference reflects the fact that a patient is stable during the vast majority of the transfer time, even if they experience a deterioration event at some point during the journey. Time windows from a given patient belong exclusively to one of the three groups.

### Model performance

**Fig 4:**
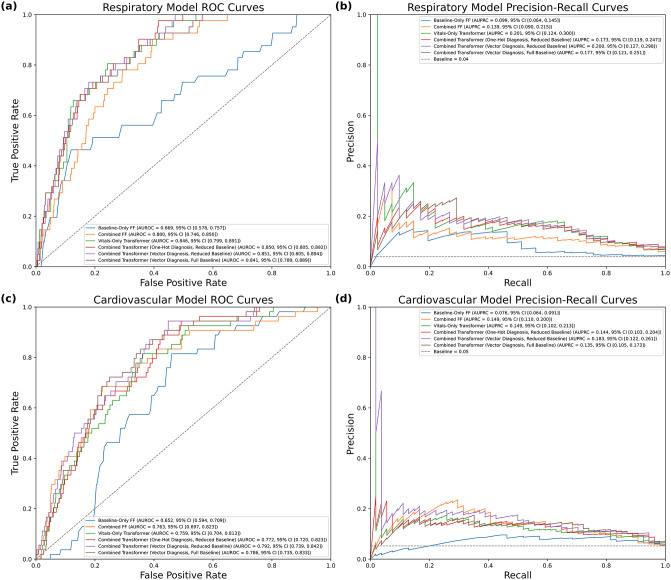
a) Stacked Receiver Operating Characteristic curves for the six respiratory. b) Stacked Precision-Recall curves for the trained respiratory models. c) Stacked ROC curves for the six cardiovascular. d) Stacked PR curves for the trained cardiovascular models. In all graphs we see that incorporating the vital sign data processing it with a transformer network boosts performance in both AUROC and AUPRC.

Performance was evaluated on the holdout test set. Complete performance metrics across all six architectures are presented in Table 4 with AUROC and AUPRC curves for each model displayed in Fig 4.

**Table 4:** Results of evaluating the model on the holdout test set.

Model	AUROC	AUPRC	Sensitivity	Specificity	PPV	NPV	Balanced Accuracy	F1-Score
**Respiratory Models**								
Baseline-Only FF	0.669 (0.578-0.757)	0.099 (0.064-0.145)	0.413 (0.268-0.561)	0.892 (0.873-0.911)	0.137 (0.090-0.185)	0.973 (0.967-0.980)	0.653 (0.579-0.729)	0.206 (0.136-0.277)
Combined FF	0.800 (0.746-0.850)	0.139 (0.090-0.215)	0.610 (0.463-0.756)	0.803 (0.778-0.828)	0.114 (0.086-0.142)	0.980 (0.973-0.988)	0.706 (0.629-0.780)	0.192 (0.145-0.237)
Vitals-Only Transformer	0.846 (0.799-0.891)	0.201 (0.124-0.300)	0.707 (0.561-0.829)	0.804 (0.779-0.828)	0.130 (0.104-0.157)	0.985 (0.978-0.991)	0.755 (0.684-0.823)	0.220 (0.177-0.263)
Combined Transformer (One-Hot Diagnosis, Reduced Baseline)	0.850 (0.805-0.892)	0.173 (0.119-0.247)	0.707 (0.561-0.829)	0.821 (0.797-0.844)	0.141 (0.112-0.170)	0.985 (0.979-0.992)	0.764 (0.693-0.832)	0.235 (0.188-0.282)
Combined Transformer (Vector Diagnosis, Reduced Baseline)	**0.851 (0.805-0.894)**	0.200 (0.127-0.298)	0.730 (0.585-0.854)	0.792 (0.766-0.817)	0.127 (0.102-0.152)	0.986 (0.979-0.993)	0.761 (0.690-0.828)	0.216 (0.174-0.257)
Combined Transformer (Vector Diagnosis, Full Baseline)	0.841 (0.789-0.889)	0.177 (0.121-0.251)	0.70 7 (0.561-0.830)	0.802 (0.777-0.826)	0.129 (0.104-0.155)	0.985 (0.978-0.992)	0.754 (0.685-0.823)	0.218 (0.176-0.261)
**Cardiovascular Models**								
Baseline-Only FF	0.652 (0.594-0.709)	0.076 (0.064-0.091)	0.037 (0.000-0.093)	0.892 (0.871-0.911)	0.019 (0.000-0.047)	0.944 (0.941-0.947)	0.464 (0.441-0.494)	0.025 (0.000-0.062)
Combined FF	0.763 (0.697-0.823)	0.149 (0.110-0.200)	0.481 (0.352-0.611)	0.833 (0.810-0.856)	0.138 (0.102-0.175)	0.967 (0.959-0.975)	0.657 (0.591-0.727)	0.214 (0.158-0.271)
Vitals-Only Transformer	0.759 (0.704-0.813)	0.149 (0.102-0.213)	0.500 (0.370-0.630)	0.822 (0.798-0.845)	0.135 (0.101-0.170)	0.968 (0.959-0.976)	0.661 (0.595-0.728)	0.212 (0.159-0.266)
Combined Transformer (One-Hot Diagnosis, Reduced Baseline)	0.772 (0.720-0.823)	0.144 (0.103-0.204)	0.463 (0.333-0.593)	0.845 (0.822-0.866)	0.142 (0.103-0.181)	0.966 (0.958-0.974)	0.654 (0.586-0.721)	0.217 (0.157-0.277)
Combined Transformer (Vector Diagnosis, Reduced Baseline)	**0.792 (0.739-0.842)**	0.183 (0.122-0.261)	0.500 (0.370-0.630)	0.855 (0.832-0.876)	0.160 (0.120-0.204)	0.969 (0.961-0.977)	0.677 (0.611-0.747)	0.242 (0.182-0.308)
Combined Transformer (Vector Diagnosis, Full Baseline)	0.786 (0.735-0.833)	0.135 (0.105-0.173)	0.501 (0.370-0.630)	0.831 (0.808-0.855)	0.141 (0.105-0.178)	0.968 (0.960-0.976)	0.666 (0.599-0.734)	0.220 (0.164-0.275)

#### Respiratory events.

Model performance for predicting respiratory deterioration significantly improved with the inclusion of time-series data. While the demographic-only baseline model showed limited predictive capability, the transition to transformer-based architectures markedly increased performance, with the Vitals-Only Transformer achieving an AUROC of 0.846. The addition of clinical diagnostic features provided marginal further gains; the best-performing configuration was the Combined Transformer (Vector Diagnosis, Reduced Baseline), which achieved an AUROC of 0.851 and an AUPRC of 0.200. Notably, the inclusion of the full set of baseline features did not offer additional predictive value, suggesting that the physiological signal is the primary driver of accuracy for respiratory forecasting.

#### Cardiovascular events.

Consistent with the respiratory models, cardiovascular prediction was weakest when relying solely on static baseline features. Predictive power increased significantly when processing continuous vital signs through transformer networks. The Combined Transformer (Vector Diagnosis, Reduced Baseline) emerged as the most robust model, yielding the highest AUROC (0.792) and AUPRC (0.183). Similar to the respiratory findings, simplified baseline feature sets (Reduced Baseline) better than configurations utilizing the full array of EHR variables, emphasizing the importance of high-frequency physiological data over sparse demographic information in this high-acuity setting.

#### Impact of historic context.

To evaluate the contribution of the upto 120-minute historical context, we compared the top-performing Combined Transformer models against baseline variants trained on only immediate 15-minute input window. The inclusion of the two-hour historical trajectory resulted in a substantial performance boost across both tasks. For respiratory deterioration, the AUROC improved from 0.794 to 0.851 and the AUPRC from 0.173 to 0.200. Similarly, cardiovascular predictive performance rose from an AUROC of 0.765 to 0.792 and an AUPRC of 0.126 to 0.183 (S8 Fig). These results indicate that the patient’s prior physiological trend provides essential context that allows the models to more accurately interpret acute shifts in vital signs.

Table 4 reports the results of evaluating the trained models for each of the two prediction tasks on the holdout test set. The decision threshold was determined on the tuning set by selecting the value that yielded a maximum false positive rate (FPR) of 20% (i.e., a minimum specificity of 80%). The values in brackets represent the 95% confidence interval calculated by performing stratified bootstrapping with replacement on the test set. Glossary of Metrics: AUROC (Area Under the Receiver Operating Characteristic curve) measures the model’s overall ability to distinguish between stable and deteriorating states across all potential decision thresholds, where a score of 1.0 is perfect and 0.5 is equivalent to random chance; AUPRC (Area Under the Precision-Recall Curve) provides a rigorous evaluation of model performance in imbalanced datasets where the event of interest is rare, focusing specifically on the quality of positive predictions; Sensitivity (Recall) is the proportion of actual deterioration events that the model correctly identified; Specificity is the proportion of stable periods that the model correctly identified as non-events; PPV (Positive Predictive Value/ Precision) is the probability that a “high risk” alert from the model truly precedes a deterioration event; NPV (Negative Predictive Value) is the probability that a “stable” prediction correctly identifies a true lack of deterioration; Balanced Accuracy is the arithmetic mean of sensitivity and specificity, providing a balanced assessment of accuracy that accounts for the rarity of events; and the F1-Score is the harmonic mean of precision and recall, serving as a single metric to evaluate the trade-off between the need to catch all clinical events and the need to minimize false alarms.

### Model explainability

#### Cohort-level feature importance.

**Fig 5:**
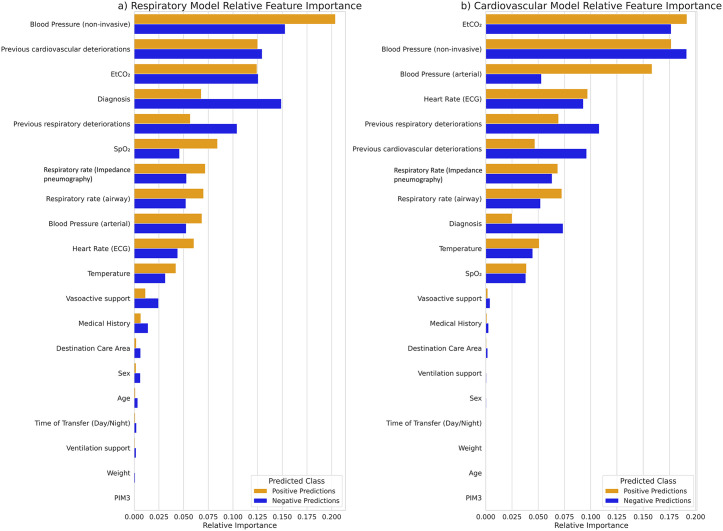
a) Relative importance of the features used by the respiratory model in predicting adverse respiratory events. b) Relative importance of the features used by the cardiovascular model in predicting cardiovascular events. Feature importance for both graphs calculated on the holdout test set.

Feature attributions for the respiratory and cardiovascular models are shown in Fig 5. In the respiratory model, the most influential features were non-invasive blood pressure, prior cardiovascular deteriorations, EtCO₂, diagnosis, and prior respiratory deteriorations. Positive predictions relied more heavily on SpO₂, while negative predictions emphasised diagnosis and prior respiratory history. For the cardiovascular model, EtCO₂, blood pressure (both non-invasive and arterial), heart rate, and prior deteriorations were dominant, with arterial pressure driving positive predictions and diagnosis driving negative ones. Overall, dynamic vital sign features contributed more to model decisions than static baseline demographics.

#### Individual-level interpretability.

**Fig 6:**
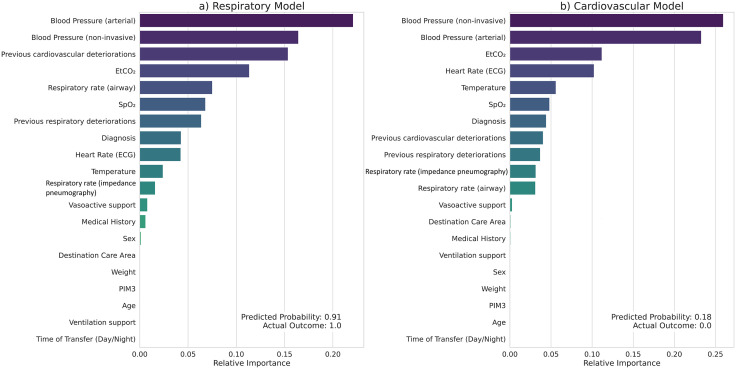
Feature importance in predictions made for a given data window in the same transport episode. **(a)** For the respiratory model, the predicted probability of an adverse respiratory event was 0.91 (and the event did occur). The most influential features were arterial and non-invasive blood pressure, previous cardiovascular deteriorations, EtCO₂, respiratory rate, SpO₂, and previous respiratory deteriorations. **(b)** For the cardiovascular model, the predicted probability of an adverse cardiovascular event was 0.18 (and the event did not occur). Non-invasive and arterial blood pressure, EtCO₂, heart rate, and temperature had the greatest impact on the prediction.

The application of this method to individual prediction windows is illustrated in Fig 6. Fig 6A shows a true-positive prediction of respiratory deterioration (probability 0.91), driven primarily by arterial and non-invasive blood pressure, EtCO₂, and respiratory rate. In contrast, Fig 6B shows a true-negative prediction for cardiovascular deterioration (probability 0.18), where the model’s assurance of stability was derived largely from blood pressure, EtCO₂, and heart rate.

#### Dynamic event correlation.

**Fig 7:**
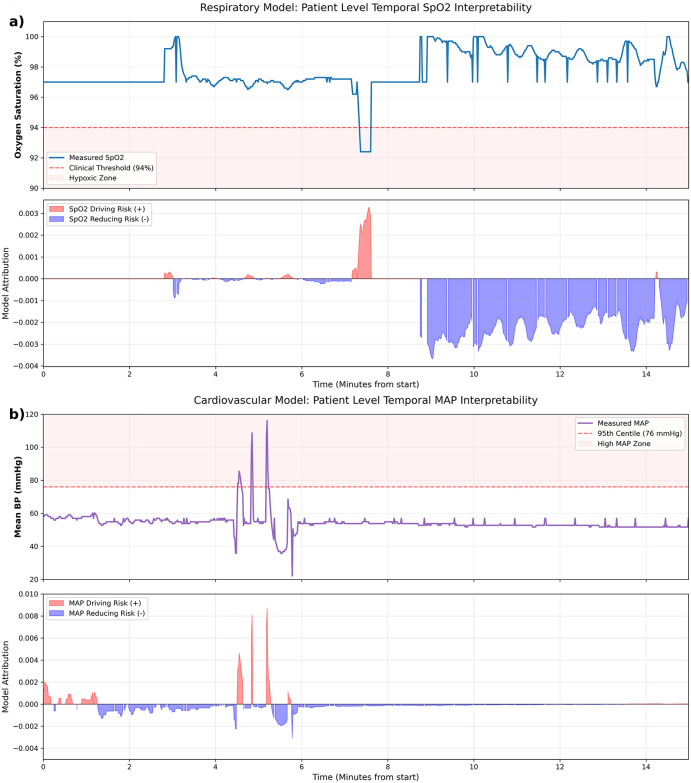
Temporal alignment of model attribution with acute clinical events. **(a)** Localized desaturation event SpO2 < 94% shown alongside a corresponding surge in “risky” model attribution (red), indicating high sensitivity to hypoxic episodes. **(b)** Model responsiveness to cumulative physiological stress; three consecutive hypertensive events (>95th centile) trigger synchronized spikes in risk attribution, demonstrating consistency with established paediatric clinical thresholds.

Further demonstration of the model’s responsiveness to clinical events is demonstrated during the transport of a 2-year-old patient in Fig 7. In Fig 7A, a localized desaturation event (SpO₂ < 94%) triggers a simultaneous spike in “risky” attribution (red), showing the model’s immediate sensitivity to hypoxia. Fig 7B demonstrates the model’s ability to identify three distinct blood pressure spikes above the 95th centile (76 mmHg) resulting in three corresponding surges in model attribution. This temporal alignment suggests that the model’s internal logic is well-synchronized with established paediatric clinical thresholds.

### Computational efficiency

Both top-performing models demonstrated high computational efficiency suitable for edge deployment. The parameter counts for the respiratory and cardiovascular models were <700,000, with a serialized memory footprint of <2.7 MB per model. When benchmarked locally on a standard laptop CPU, the average inference latency was < 60 ms per 15-minute data window.

## Discussion

To our knowledge, this is the first study to use continuously collected vital signs and linked EHR data during interhospital transport of critically ill children to predict real-time risk of deterioration. We present two lightweight, explainable machine learning models that integrate live-streamed physiology with baseline clinical data to forecast respiratory and cardiovascular events up to 15 minutes in advance, potentially enabling earlier intervention and preventing decline.

Previous studies have largely focused on predicting mortality during transport or deterioration in stable, non-critically ill paediatric inpatients [11,13,22–24]. While several of these models have demonstrated strong performance in predicting ward-to-PICU transfers or ICU mortality, none have been specifically developed to predict imminent deterioration in critically ill children during interhospital transport. Our work addresses this gap, introducing a real-time risk prediction tool for one of the most high-risk settings in paediatric care.

The models were developed using a diverse dataset of over 1,500 interhospital transports conducted by the CATS team in central London. We adopted a systematic approach, starting with models based solely on demographic and pre-transport features, then incorporating high-resolution time-series data. Initial models used simple feed-forward networks, progressing to transformer-based architectures for more advanced temporal modelling. We also evaluated multiple strategies for diagnosis representation, comparing one-hot encoding against vector embeddings derived from a pretrained clinical language model [17]. This iterative, constructive approach allowed us to quantify how predictive performance scaled with increasing model complexity and feature richness, while identifying the minimal configuration needed to maintain high accuracy. High-frequency vital-sign data, particularly when processed using transformer-based models capable of capturing complex temporal dynamics significantly boosted accuracy.

Our best-performing models for both tasks used the Combined Transformer (Vector Diagnosis, Reduced Baseline) architecture. On the holdout test set, the respiratory deterioration model achieved an AUROC of 0.851 and AUPRC of 0.200, with a sensitivity of 0.730 and specificity of 0.792. The cardiovascular model yielded an AUROC of 0.792 and AUPRC of 0.183, with a sensitivity of 0.500 and specificity of 0.855. These results are particularly notable given the low baseline incidence of events in the test set: only 3.1% of time windows included respiratory deterioration, 4.4% included cardiovascular deterioration, and just 0.87% involved both. This extreme class imbalance underscores the models’ ability to extract clinically salient signals from noisy transport data.

Beyond overall performance, we used Integrated Gradients to understand how the best-performing models made predictions, assessing feature importance at both the cohort and individual level [21]. In the respiratory model, the top contributors were non-invasive blood pressure, prior cardiovascular events, and EtCO₂, followed by diagnosis, prior respiratory events, and SpO₂, likely reflecting the interdependent nature of respiratory and cardiovascular physiology, where cardiovascular instability is often associated with respiratory instability. The cardiovascular model similarly emphasised EtCO₂, non-invasive and arterial blood pressure, heart rate and previous episodes of respiratory or cardiovascular instability. The high weighting of EtCO₂ may again reflect the interdependent nature of respiratory and cardiovascular physiology, where CO2 is not only a marker of ventilation but also perfusion [25]. Furthermore, the presence of invasive blood pressure, available in ~36% of transport episodes, was associated with positive predictions, likely reflecting the fact that patients requiring arterial lines are more haemodynamically unstable and thus at higher risk of deterioration. Interestingly, diagnosis was more influential in negative predictions, suggesting the model learned that certain conditions confer lower deterioration risk. These insights highlight the models’ ability to provide interpretable outputs that align with clinical reasoning, a key factor in promoting clinician trust and potential real-world adoption. From a clinical interface perspective, these feature attributions could be translated into real-time “explainable alerts” on transport monitors. Rather than presenting a solitary risk probability, the system could dynamically flag the top-contributing physiological parameters; for example, explicitly indicating that a rising respiratory risk score is being driven by specific instabilities in EtCO2 and SpO2. Such an approach allows clinicians to rapidly cross-reference the AI’s logic with their own clinical assessment, facilitating more targeted interventions and reducing the cognitive load associated with interpreting raw high-frequency data in a high-pressure environment. By transforming this high-frequency data into an actionable 15-minute lead time, the model enables a shift from reactive crisis management to proactive stabilization. For example, a rising respiratory risk attribution could prompt a clinician to perform a focused assessment of the endotracheal tube position or optimize recruitment manoeuvrers before a significant desaturation occurs, ultimately facilitating smoother, safer transitions of care.

A further key strength of our models is their computational efficiency and suitability for real-time deployment in resource-constrained settings. Both are lightweight, with parameter counts below 700,000 and a serialized memory footprint of less than 2.7 MB, making them ideal for the edge devices used by transport teams. Benchmark testing conducted locally on a consumer grade laptop (Intel Core i7) without remote server access or GPU acceleration demonstrated that both models generate predictions in under 60 ms per 15-minute window. This performance supports near-instantaneous real-time risk assessment on standard clinical hardware. Integrated Gradients, used for interpretability, is similarly efficient and can run in real-time on the same device, providing patient-specific explanations alongside risk scores. Crucially, all computations are performed locally, ensuring that decision support remains available in transport environments where cellular or satellite connectivity may be intermittent. Together, these features make real-world deployment in paediatric critical care transport both practical and feasible.

One limitation is the availability and continuity of monitoring data: only 23.7% (1,519 of 6,471) of transports met inclusion criteria. However, included cases closely matched the overall cohort across key demographic and clinical variables. It is important to clarify that the limited data capture was a result of research-specific logistical constraints rather than clinical selection; while all patients were continuously monitored, only two of three transport teams were equipped with the hardware necessary to transmit high-frequency data to a secure research server. Because device assignment was determined by vehicle rotation and hardware availability rather than patient acuity, the risk of selection bias was minimized. This is substantiated by our analysis, which demonstrates that the included cohort remained demographically and clinically representative of the total transport population (n = 6,471) across age, diagnosis, and illness severity. Additionally, we observed high rates of missingness in several demographic fields, most notably ethnicity (51.7%). While our sensitivity analysis revealed that a reduced feature set (excluding these sparse demographic variables) improved model performance by focusing on high-density physiological signals, we acknowledge that the exclusion of sensitive attributes does not fully insulate a model from bias. Clinical data often contains embedded systemic inequities that machine learning can inadvertently learn. In this retrospective study, a robust subgroup analysis for fairness was not statistically viable due to the highly fragmented distribution of ethnicity in the holdout test set (n = 155), where 79 patients had unknown ethnicity and several minority categories were represented by only a single patient. Evaluating performance on such small subgroups would yield unstable and potentially misleading results.These findings suggest that while the immediate physiological trajectory is a more robust predictor of acute deterioration than static baseline characteristics, achieving complete data capture in emergency environments remains an ongoing challenge. Proof-of-concept studies like this are essential to advocate for the future expansion of clinical data infrastructure and larger, more diverse cohorts where powered, pre-specified subgroup analyses can appropriately evaluate model performance across all demographic backgrounds.

Furthermore, although our models achieved strong discrimination with high AUROCs, their positive predictive values (PPVs) and F1-scores were modest; largely due to the low prevalence of adverse events (4–5% of windows). As a result, even with high specificity, false positives outnumber true positives, leading to lower PPV and F1-scores. This challenge is not unique to our study and is a recognised limitation in predictive modelling for rare but critical events. Similar findings have been reported in other early warning systems, where models predicting rare but critical deterioration events (such as ICU transfers, in-hospital cardiac arrests, or ward-based decompensation) achieved high AUROCs (>0.85) but relatively low PPVs (typically ~10–15%), due to the low event prevalence [26–28]. To mitigate the risk of alarm fatigue resulting from these modest PPVs, we intentionally chose a decision threshold on the tuning set that enforced a minimum specificity of 80% (a maximum False Positive Rate of 20%). Despite appearing suboptimal in absolute terms, such PPVs can still be clinically acceptable, particularly in the absence of alternative systems or when coupled with high sensitivity and fewer false alarms than traditional scoring methods. Crucially, we do not envision these models as autonomous diagnostic tools, but rather as adjunctive digital safety nets designed to complement, not replace, clinical judgment. The specific probability threshold that should justify intervention is not a “one-size-fits-all” value; rather, it is highly context-dependent and likely to vary based on regional protocols, patient acuity, and the clinician’s experience. By providing a customizable and explainable output, the model allows practitioners to calibrate the “digital nudge” to their specific operational tolerance. Further testing in prospective trials should include detailed examination of human factors and clinician preferences in managing the balance between sensitivity and false alarm rates, eventually establishing how these risk magnitudes should be integrated into standardized clinical escalation pathways.

Looking ahead, external validation is essential before real-world deployment. Testing on datasets from other paediatric transport services (both nationally and internationally) will be crucial to assess generalisability across different patient populations, transport systems, monitoring technologies, and clinical practices. Multicentre validation would also enable retraining or fine-tuning on more heterogeneous data, improving robustness and broader applicability. Following this, prospective deployment studies will be needed to evaluate real-world performance, clinical decision-making and ultimately, patient outcomes. Furthermore, the general framework presented here could be adapted to in-hospital PICU applications relating to risk of clinical deterioration. Here, similar models could serve as adjunctive tools to support continuous risk stratification and enable proactive intervention in children already receiving intensive care.

## Conclusion

We present the first real-time machine learning models capable of predicting acute deterioration in critically ill children during interhospital transport using routinely collected high-frequency vital sign and clinical data. These lightweight, explainable models perform well and can run on edge devices, making them practical for resource-limited settings. Our findings suggest significant

### Code availability

All code and trained models associated with this project are available at https://github.com/MilanKapur1/paediatric_tranport_deterioration_prediction.

## Supporting information

S1 Fig Histogram of duration of each of the 1519 included transport episodes. The dashed red line marks the median duration with the grey shaded region marking the inter-quartile range (IQR: 75–160 minutes).
(DOCX)

S1 Table Summary of the key characteristics of the predictive models developed to independently forecast adverse respiratory and cardiovascular events within a 15-minute window. The table descends in order of first computational complexity and then feature complexity. Transformer blocks were implemented rotary positional embeddings, and a decoder-only setup that ensures one-directional (causal) attention for time-series data. The table is ordered to follow incremental progression in model complexity and input feature detail.
(DOCX)

S2 Fig Architecture of the baseline-only feed-forward model. a) Architecture for respiratory model. b) Architecture for cardiovascular model. Each model consists of two parallel feed-forward branches: one processes all pre-transport baseline features (including patient demographics, transport details, pre-transport interventions, and intra-transport support), while the other processes the vector-embedded primary diagnosis. The outputs of both branches are concatenated and passed through a final feed-forward network to generate the prediction.
(DOCX)

S3 Fig Architecture of the combined feed-forward model. a) Architecture for respiratory model. b) Architecture for cardiovascular model. Each model comprises three parallel feed-forward branches: one processes vital signs and pre-occurring adverse events, another handles a reduced subset of baseline features (including age, weight, sex, PIM3 score, destination care area, pre-existing medical conditions, and intra-transport support), and the third processes the vector-embedded primary diagnosis. Outputs from all three branches are concatenated and passed through a final feed-forward network to generate the prediction.
(DOCX)

S4 Fig Architecture of the vitals-only transformer model. a) Architecture for respiratory model. b) Architecture for cardiovascular model. Each model consists of a single transformer branch that processes time-series vital signs and any pre-occurring adverse events. The output of the transformer is passed through a final feed-forward layer to generate the prediction. No baseline demographic or diagnostic information is included.
(DOCX)

S5 Fig Architecture of the combined transformer (One-Hot diagnosis, reduced baseline) model. a) Architecture for respiratory model. b) Architecture for cardiovascular model. Each model integrates two branches: a transformer for processing time-series vital signs, and a feed-forward network for a reduced subset of baseline features (including age, weight, sex, PIM3 score, destination care area, pre-existing medical conditions, intra-transport support, and one-hot encoded primary diagnosis). Outputs from both branches are concatenated and passed through a final feed-forward network to generate the prediction.
(DOCX)

S6 Fig Architecture of the Combined Transformer (Vector Diagnosis, Reduced Baseline) model. a) Architecture for respiratory model. b) Architecture for cardiovascular model. Each model integrates three branches: a transformer for processing time-series vital signs, a feed-forward network for a reduced subset of baseline features (including age, weight, sex, PIM3 score, destination care area, pre-existing medical conditions, and intra-transport support), and a separate feed-forward network for the vector-embedded primary diagnosis. Outputs from all branches are concatenated and passed through a final feed-forward network to generate the prediction.
(DOCX)

S7 Fig Architecture of the Combined Transformer (Vector Diagnosis, Full Baseline) model. a) Architecture for respiratory model. b) Architecture for cardiovascular model. Each model integrates three branches: a transformer for processing time-series vital signs, a feed-forward network for the full set of baseline features (including age, weight, sex, ethnicity, pre-transport interventions, and intra-transport support), and a separate feed-forward network for vector-embedded primary diagnosis. Outputs from all branches are concatenated and passed through a final feed-forward network to generate the prediction.
(DOCX)

S8 Fig Study evaluating the impact of historical physiological context. Performance curves for models trained without the 120-minute historical context (utilizing only the immediate 15-minute high-resolution window). (a) Receiver Operating Characteristic (ROC) curve for the respiratory model. (b) Precision-Recall (PR) curve for the respiratory model. (c) ROC curve for the cardiovascular model. (d) PR curve for the cardiovascular model. Comparisons with the primary results (Table 4) demonstrate that excluding the historical context leads to a marked decrease in both discriminative power (AUROC) and precision (AUPRC).
(DOCX)

## References

[1] Paediatric Intensive Care Audit Network (PICANet). National paediatric critical care audit state of the nation report 2024. Healthcare Quality Improvement Partnership (HQIP); 2024. https://www.picanet.org.uk/annual-reporting-and-publications/

[2] Orr RA, Felmet KA, Han Y, McCloskey KA, Dragotta MA, Bills DM, et al. Pediatric specialized transport teams are associated with improved outcomes. Pediatrics. 2009;124(1):40–8. doi: 10.1542/peds.2008-0515 19564281

[3] Singh JM, Gunz AC, Dhanani S, Aghari M, MacDonald RD. Frequency, composition, and predictors of in-transit critical events during pediatric critical care transport*. Pediatric Critical Care Med. 2016;17(10):984. doi: 10.1097/PCC.0000000000000919 27505717

[4] Haydar B, Baetzel A, Elliott A, MacEachern M, Kamal A, Christensen R. Adverse events during intrahospital transport of critically Ill children: a systematic review. Anesth Analg. 2020;131(4):1135–45. doi: 10.1213/ANE.0000000000004585 32925334

[5] Huo Z, Booth J, Monks T, Knight P, Watson L, Peters M, et al. Distribution and trajectory of vital signs from high-frequency continuous monitoring during pediatric critical care transport. Intensive Care Med Paediatr Neonatal. 2023;1(1). doi: 10.1007/s44253-023-00018-x

[6] Fleming S, Thompson M, Stevens R, Heneghan C, Plüddemann A, Maconochie I, et al. Normal ranges of heart rate and respiratory rate in children from birth to 18 years of age: a systematic review of observational studies. Lancet. 2011;377(9770):1011–8. doi: 10.1016/S0140-6736(10)62226-X 21411136 PMC3789232

[7] Bonafide CP, Brady PW, Keren R, Conway PH, Marsolo K, Daymont C. Development of heart and respiratory rate percentile curves for hospitalized children. Pediatrics. 2013;131(4):e1150-7. doi: 10.1542/peds.2012-2443 23478871 PMC4074640

[8] Nielsen VML, Kløjgård T, Bruun H, Søvsø MB, Christensen EF. Progression of vital signs during ambulance transport categorised by a paediatric triage model: a population-based historical cohort study. BMJ Open. 2020;10(11):e042401. doi: 10.1136/bmjopen-2020-042401 33257494 PMC7705491

[9] Kapur M, Li K, Brown A, Huo Z, Knight P, Davies G, et al. Identification of physiological adverse events using continuous vital signs monitoring during paediatric critical care transport: a novel data-driven approach. PLOS Digit Health. 2025;4(9):e0000822. doi: 10.1371/journal.pdig.0000822 40997103 PMC12463272

[10] Shamout FE, Shen Y, Wu N, Kaku A, Park J, Makino T, et al. An artificial intelligence system for predicting the deterioration of COVID-19 patients in the emergency department. NPJ Digit Med. 2021;4(1):80. doi: 10.1038/s41746-021-00453-0 33980980 PMC8115328

[11] Huo Z, Booth J, Monks T, Knight P, Watson L, Peters M, et al. Dynamic mortality prediction in critically Ill children during interhospital transports to PICUs using explainable AI. NPJ Digit Med. 2025;8(1):108. doi: 10.1038/s41746-025-01465-w 39962177 PMC11832768

[12] Sundrani S, Chen J, Jin BT, Abad ZSH, Rajpurkar P, Kim D. Predicting patient decompensation from continuous physiologic monitoring in the emergency department. NPJ Digit Med. 2023;6(1):60. doi: 10.1038/s41746-023-00803-0 37016152 PMC10073111

[13] Jeon Y, Kim YS, Jang W, Park JD, Lee B. Development of a deep learning model that predicts critical events of pediatric patients admitted to general wards. Sci Rep. 2024;14(1):4707. doi: 10.1038/s41598-024-55528-1 38409469 PMC10897152

[14] Victor A. The role of artificial intelligence in maternal and child health: progress, controversies, and future directions. PLOS Digit Health. 2025;4(7):e0000938. doi: 10.1371/journal.pdig.0000938 40674369 PMC12270093

[15] Straney L, Clements A, Parslow RC, Pearson G, Shann F, Alexander J, et al. Paediatric index of mortality 3: an updated model for predicting mortality in pediatric intensive care*. Pediatr Crit Care Med. 2013;14(7):673–81. doi: 10.1097/PCC.0b013e31829760cf 23863821

[16] NHS Digital. Data quality of protected characteristics and other vulnerable groups: ethnicity. NHS England Digital. https://digital.nhs.uk/data-and-information/data-collections-and-data-sets/data-sets/mental-health-services-data-set/submit-data/data-quality-of-protected-characteristics-and-other-vulnerable-groups/ethnicity

[17] Alsentzer E, Murphy J, Boag W, et al. Publicly available clinical BERT embeddings. In: Proceedings of the 2nd clinical natural language processing workshop, 2019. 72–8. doi: 10.18653/v1/W19-1909

[18] Kapur M, Li K, Brown A. Identification of physiological adverse events using continuous vital signs monitoring during paediatric critical care transport: a novel data-driven approach. medRxiv. 2025. doi: 10.1101/2025.03.11.25323742 PMC12463272 40997103

[19] Vaswani A, Shazeer N, Parmar N. Attention is all you need. arXiv. 2023;1706.03762.doi: 10.48550/arXiv.1706.03762

[20] Su J, Ahmed M, Lu Y, Pan S, Bo W, Liu Y. RoFormer: enhanced transformer with rotary position embedding. Neurocomput. 2024;568(C). doi: 10.1016/j.neucom.2023.127063

[21] Sundararajan M, Taly A, Yan Q. Axiomatic attribution for deep networks. arXiv. 2017. doi: 10.48550/arXiv.1703.01365

[22] Mayampurath A, Jani P, Dai Y, Gibbons R, Edelson D, Churpek MM. A vital sign-based model to predict clinical deterioration in hospitalized children. Pediatr Crit Care Med. 2020;21(9):820–6. doi: 10.1097/PCC.0000000000002414 32511200 PMC7483876

[23] Rust LOH, Gorham TJ, Bambach S, Bode RS, Maa T, Hoffman JM, et al. The deterioration risk index: developing and piloting a machine learning algorithm to reduce pediatric inpatient deterioration. Pediatr Crit Care Med. 2023;24(4):322–33. doi: 10.1097/PCC.0000000000003186 36735282

[24] Early risk prediction of pediatric cardiac arrest from electronic health records via multimodal fused transformer. Accessed 2025 April 7. https://arxiv.org/html/2502.07158v2 10.1109/EMBC58623.2025.11251673 41336090

[25] Gavelli F, Teboul J-L, Monnet X. How can CO2-derived indices guide resuscitation in critically ill patients?. J Thorac Dis. 2019;11(Suppl 11):S1528–37. doi: 10.21037/jtd.2019.07.10 31388457 PMC6642918

[26] Steitz BD, McCoy AB, Reese TJ, Liu S, Weavind L, Shipley K, et al. Development and validation of a machine learning algorithm using clinical pages to predict imminent clinical deterioration. J Gen Intern Med. 2024;39(1):27–35. doi: 10.1007/s11606-023-08349-3 37528252 PMC10817885

[27] Kwon J-M, Lee Y, Lee Y, Lee S, Park J. An algorithm based on deep learning for predicting in-hospital cardiac arrest. J Am Heart Assoc. 2018;7(13):e008678. doi: 10.1161/JAHA.118.008678 29945914 PMC6064911

[28] Churpek MM, Carey KA, Snyder A, Winslow CJ, Gilbert E, Shah NS, et al. Multicenter development and prospective validation of eCARTv5: a gradient-boosted machine-learning early warning score. Crit Care Explor. 2025;7(4):e1232. doi: 10.1097/CCE.0000000000001232 40138535 PMC11949291

