# Supplementary material for: Real-time prediction of cardiorespiratory deterioration during paediatric critical care transport using interpretable machine learning
Source: PLOS Digit Health. 2026 May 19;5(5):e0001410. doi: 10.1371/journal.pdig.0001410 (PMC13186380; doi:10.1371/journal.pdig.0001410)
Supplement: S1 Fig — The dashed red line marks the median duration with the grey shaded region marking the inter-quartile range (IQR: 75–160 minutes). (DOCX) [file pdig.0001410.s001.docx]

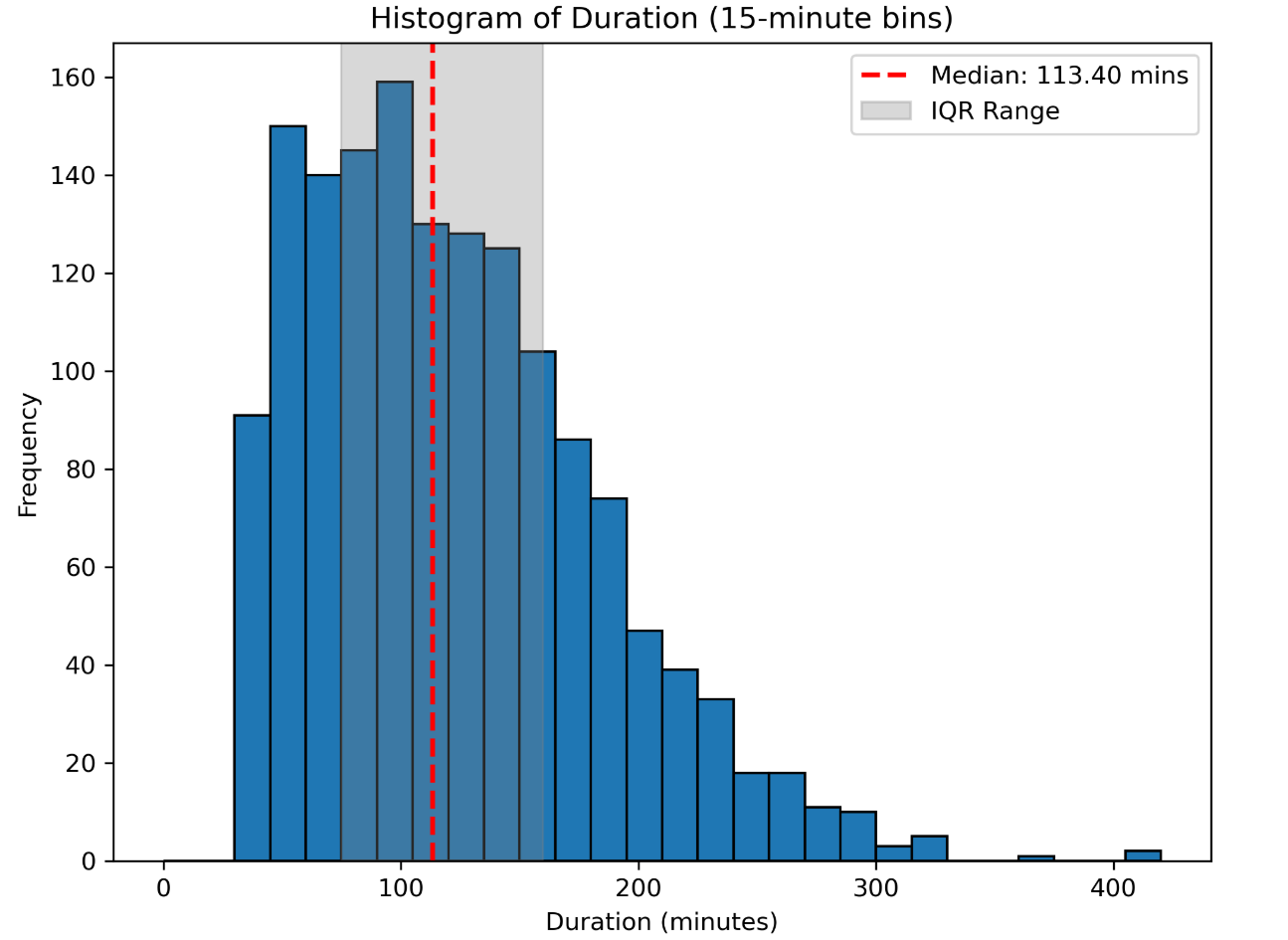


Supplementary Figure 1: Histogram of duration of each of the 1519 included transport episodes. The dashed red line marks the median duration with the grey shaded region marking the inter-quartile range (IQR: 75-160 minutes).
