# Supplementary material for: Real-time prediction of cardiorespiratory deterioration during paediatric critical care transport using interpretable machine learning
Source: PLOS Digit Health. 2026 May 19;5(5):e0001410. doi: 10.1371/journal.pdig.0001410 (PMC13186380; doi:10.1371/journal.pdig.0001410)
Supplement: S1 Table — The table descends in order of first computational complexity and then feature complexity. Transformer blocks were implemented rotary positional embeddings, and a decoder-only setup that ensures one-directional (causal) attention for time-series data. The table is ordered to follow incremental progression in model complexity and input feature detail. (DOCX) [file pdig.0001410.s002.docx]

| **Model Name** | **Model Description** | **Input Features** | **Architecture details** | **Schematic Reference** |
| --- | --- | --- | --- | --- |
| Baseline-Only FF | Baseline Features Only | All pre-transport baseline features present in Table 2: patient demographics, transport details, diagnosis (vector-embedded), interventions by the local team prior to transport, intra-transport respiratory and cardiovascular support commenced prior to transport | Two-armed architecture: One feed-forward branch handles baseline features, and a second feed-forward branch separately processes the diagnosis embedding. The outputs from both branches are then merged and passed through a final feed-forward network to generate the prediction. | Supplementary Figure 2 |
| Combined FF | Combined features: Simple Neural Network | Vital signs, associated adverse events and reduced subset of baseline features present in Table 2: age, weight, sex, PIM3, destination care area, diagnosis (vector-embedded), pre-existing medical conditions, intra-transport respiratory and cardiovascular support commenced prior to transport | Three-armed architecture: One feed-forward branch process vital signs, a second feed-forward branch handles baseline features, and a third feed-forward branch separately processes the diagnosis embedding. The outputs from all three branches are then merged and passed through a final feed-forward network to generate the prediction. | Supplementary Figure 3 |
| Vitals-Only Transformer | Vital signs only | Vital signs and pre-occurring adverse events | Transformer-based architecture: Time-series data is first processed by a transformer network, and the resulting outputs are passed through a simple feed-forward layer to generate the final prediction. | Supplementary Figure 4 |
| Combined Transformer (One-Hot Diagnosis, Reduced Baseline) | Combined features: Transformer processing of vital signs, one-hot encoded diagnosis group, reduced subset baseline features | Vital signs, associated adverse events and reduced subset of baseline features present in Table 2: age, weight, sex, PIM3, destination care area, diagnosis (one-hot encoded), pre-existing medical conditions, intra-transport respiratory and cardiovascular support commenced prior to transport | Dual-armed architecture: One branch uses a transformer to process time-series vital signs, while a separate feed-forward branch handles baseline features. The outputs of both branches are merged and passed through a final feed-forward network to produce the prediction. | Supplementary Figure 5 |
| Combined Transformer (Vector Diagnosis, Reduced Baseline) | Combined features: Transformer processing of vital signs, vector embedded diagnosis group, reduced subset baseline features | Vital signs, associated adverse events and reduced subset of baseline features present in Table 2: age, weight, sex, PIM3, destination care area, diagnosis (vector-embedded), pre-existing medical conditions, intra-transport respiratory and cardiovascular support commenced prior to transport | Three-armed architecture: One branch uses a transformer to process time-series vital signs, a second feed-forward branch handles baseline features, and a third feed-forward branch separately processes the diagnosis embedding. The outputs from all three branches are then merged and passed through a final feed-forward network to generate the prediction. | Supplementary Figure 6 |
| Combined Transformer (Vector Diagnosis, Full Baseline) | Combined features: Transformer processing of vital signs, vector embedded diagnosis group, full baseline features | Vital signs, associated adverse events and all baseline features present in Table 2: patient demographics, transport details, diagnosis (vector-embedded), interventions by the local team prior to transport, intra-transport respiratory and cardiovascular support commenced prior to transport | Three-armed architecture: One branch uses a transformer to process time-series vital signs, a second feed-forward branch handles baseline features, and a third feed-forward branch separately processes the diagnosis embedding. The outputs from all three branches are then merged and passed through a final feed-forward network to generate the prediction. | Supplementary Figure 7 |

Supplementary Table1: Description of the models developed for predicting adverse respiratory and cardiovascular events

Supplementary Table 1: Summary of the key characteristics of the predictive models developed to independently forecast adverse respiratory and cardiovascular events within a 15-minute window. The table descends in order of first computational complexity and then feature complexity. Transformer blocks were implemented rotary positional embeddings, and a decoder-only setup that ensures one-directional (causal) attention for time-series data. The table is ordered to follow incremental progression in model complexity and input feature detail.
