# Supplementary material for: Real-time prediction of cardiorespiratory deterioration during paediatric critical care transport using interpretable machine learning
Source: PLOS Digit Health. 2026 May 19;5(5):e0001410. doi: 10.1371/journal.pdig.0001410 (PMC13186380; doi:10.1371/journal.pdig.0001410)
Supplement: S2 Fig — a) Architecture for respiratory model. b) Architecture for cardiovascular model. Each model consists of two parallel feed-forward branches: one processes all pre-transport baseline features (including patient demographics, transport details, pre-transport interventions, and intra-transport support), while the other processes the vector-embedded primary diagnosis. The outputs of both branches are concatenated and passed through a final feed-forward network to generate the prediction. (DOCX) [file pdig.0001410.s003.docx]

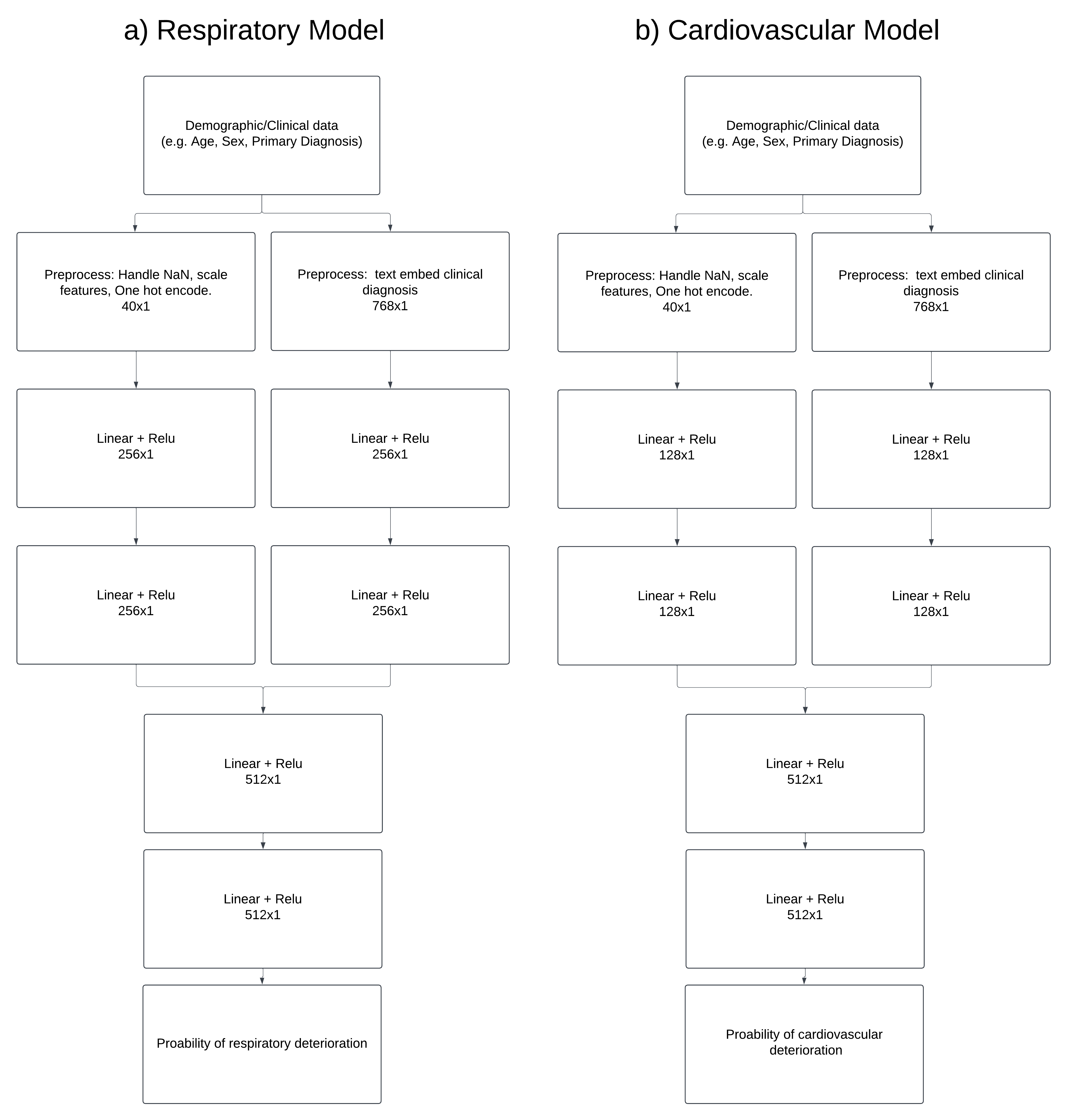


Supplementary Figure 2: Architecture of the Baseline-Only Feed-Forward model. a) Architecture for respiratory model. b) Architecture for cardiovascular model. Each model consists of two parallel feed-forward branches: one processes all pre-transport baseline features (including patient demographics, transport details, pre-transport interventions, and intra-transport support), while the other processes the vector-embedded primary diagnosis. The outputs of both branches are concatenated and passed through a final feed-forward network to generate the prediction.
