# Supplementary material for: Real-time prediction of cardiorespiratory deterioration during paediatric critical care transport using interpretable machine learning
Source: PLOS Digit Health. 2026 May 19;5(5):e0001410. doi: 10.1371/journal.pdig.0001410 (PMC13186380; doi:10.1371/journal.pdig.0001410)
Supplement: S3 Fig — a) Architecture for respiratory model. b) Architecture for cardiovascular model. Each model comprises three parallel feed-forward branches: one processes vital signs and pre-occurring adverse events, another handles a reduced subset of baseline features (including age, weight, sex, PIM3 score, destination care area, pre-existing medical conditions, and intra-transport support), and the third processes the vector-embedded primary diagnosis. Outputs from all three branches are concatenated and passed through a final feed-forward network to generate the prediction. (DOCX) [file pdig.0001410.s004.docx]

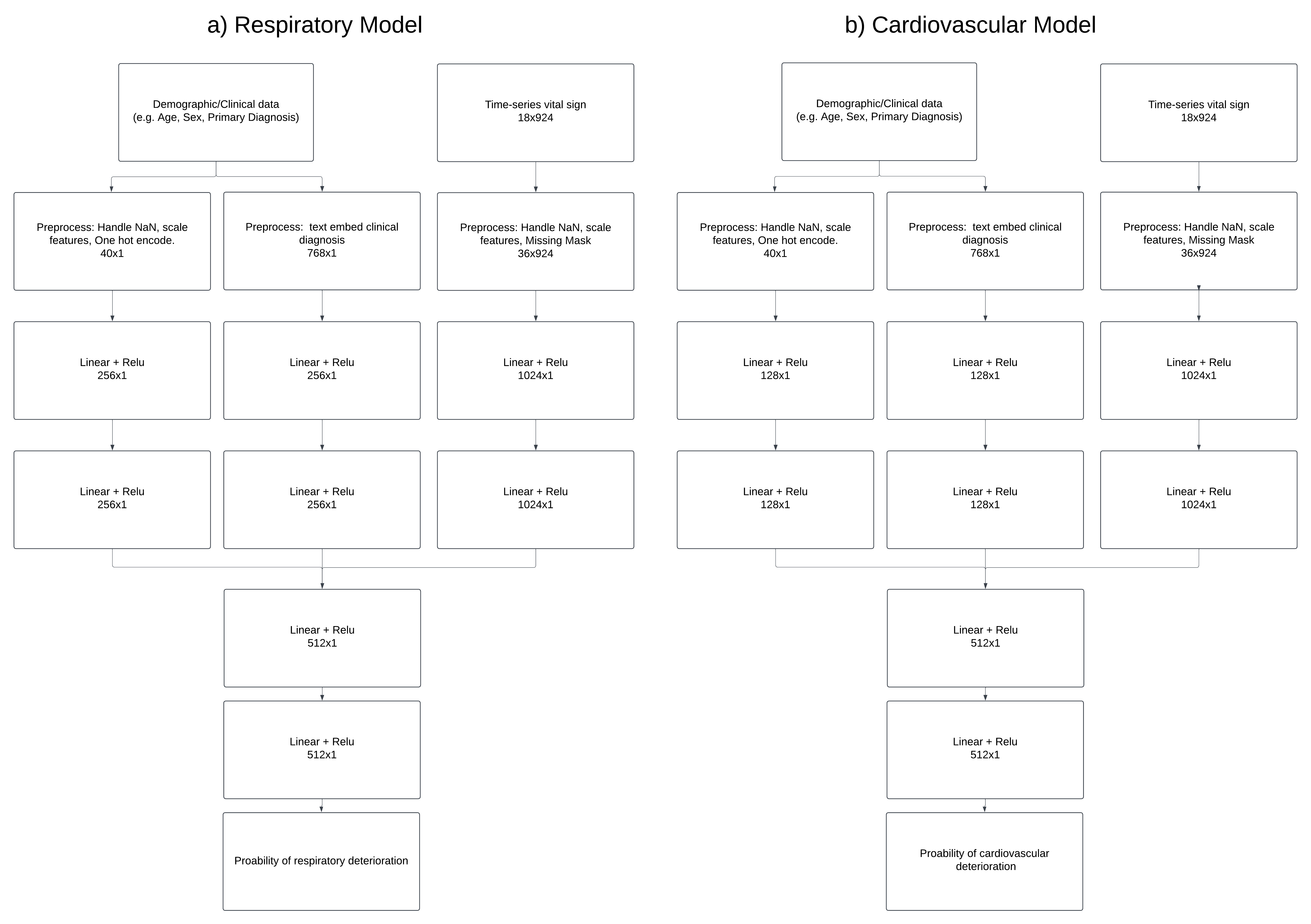


Supplementary Figure 3: Architecture of the Combined Feed-Forward model. a) Architecture for respiratory model. b) Architecture for cardiovascular model.
Each model comprises three parallel feed-forward branches: one processes vital signs and pre-occurring adverse events, another handles a reduced subset of baseline features (including age, weight, sex, PIM3 score, destination care area, pre-existing medical conditions, and intra-transport support), and the third processes the vector-embedded primary diagnosis. Outputs from all three branches are concatenated and passed through a final feed-forward network to generate the prediction.
