# Supplementary material for: Real-time prediction of cardiorespiratory deterioration during paediatric critical care transport using interpretable machine learning
Source: PLOS Digit Health. 2026 May 19;5(5):e0001410. doi: 10.1371/journal.pdig.0001410 (PMC13186380; doi:10.1371/journal.pdig.0001410)
Supplement: S4 Fig — a) Architecture for respiratory model. b) Architecture for cardiovascular model. Each model consists of a single transformer branch that processes time-series vital signs and any pre-occurring adverse events. The output of the transformer is passed through a final feed-forward layer to generate the prediction. No baseline demographic or diagnostic information is included. (DOCX) [file pdig.0001410.s005.docx]

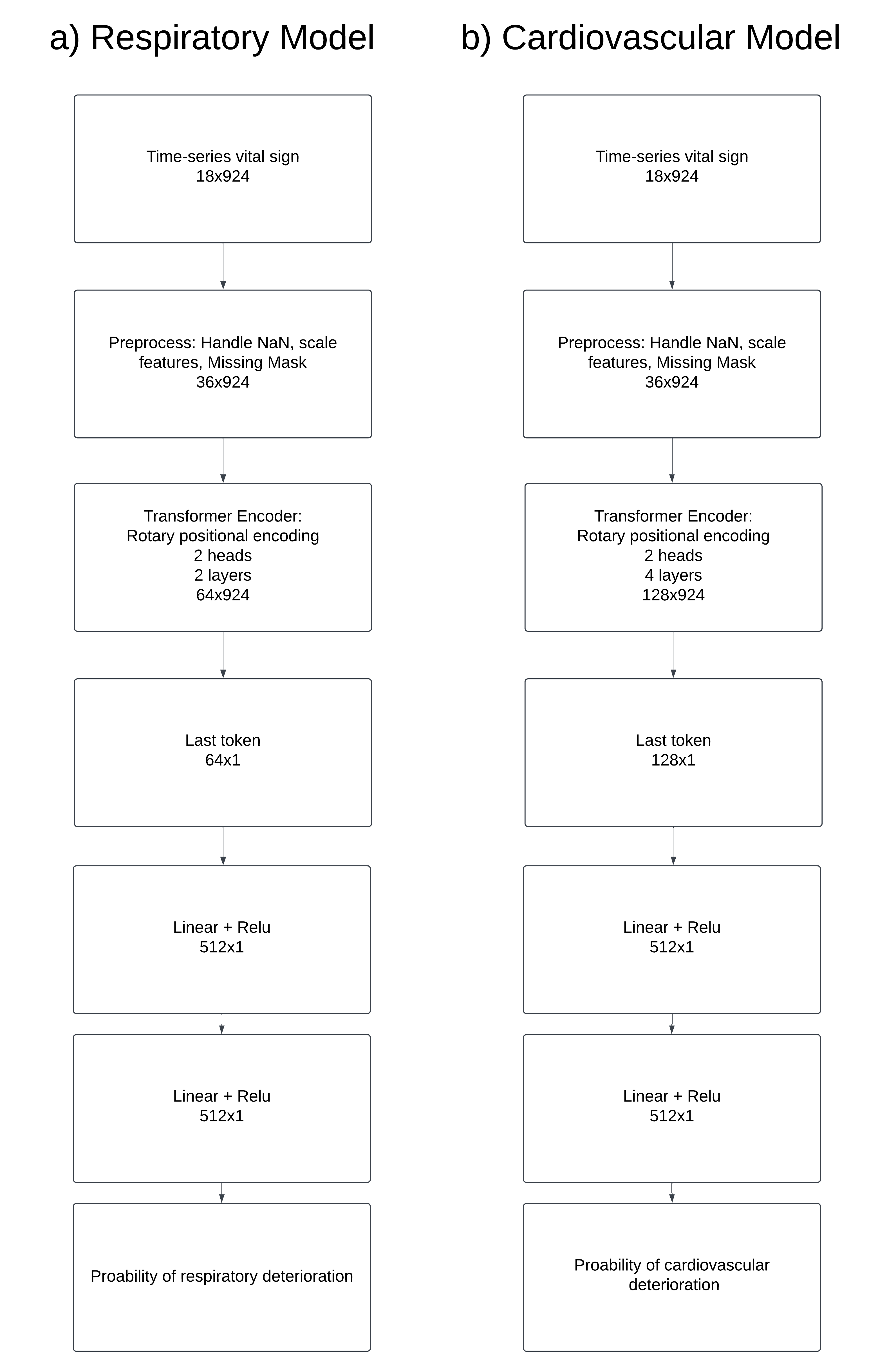


Supplementary Figure 4: Architecture of the Vitals-Only Transformer model. a) Architecture for respiratory model. b) Architecture for cardiovascular model. Each model consists of a single transformer branch that processes time-series vital signs and any pre-occurring adverse events. The output of the transformer is passed through a final feed-forward layer to generate the prediction. No baseline demographic or diagnostic information is included.
