# Supplementary material for: Real-time prediction of cardiorespiratory deterioration during paediatric critical care transport using interpretable machine learning
Source: PLOS Digit Health. 2026 May 19;5(5):e0001410. doi: 10.1371/journal.pdig.0001410 (PMC13186380; doi:10.1371/journal.pdig.0001410)
Supplement: S5 Fig — a) Architecture for respiratory model. b) Architecture for cardiovascular model. Each model integrates two branches: a transformer for processing time-series vital signs, and a feed-forward network for a reduced subset of baseline features (including age, weight, sex, PIM3 score, destination care area, pre-existing medical conditions, intra-transport support, and one-hot encoded primary diagnosis). Outputs from both branches are concatenated and passed through a final feed-forward network to generate the prediction. (DOCX) [file pdig.0001410.s006.docx]

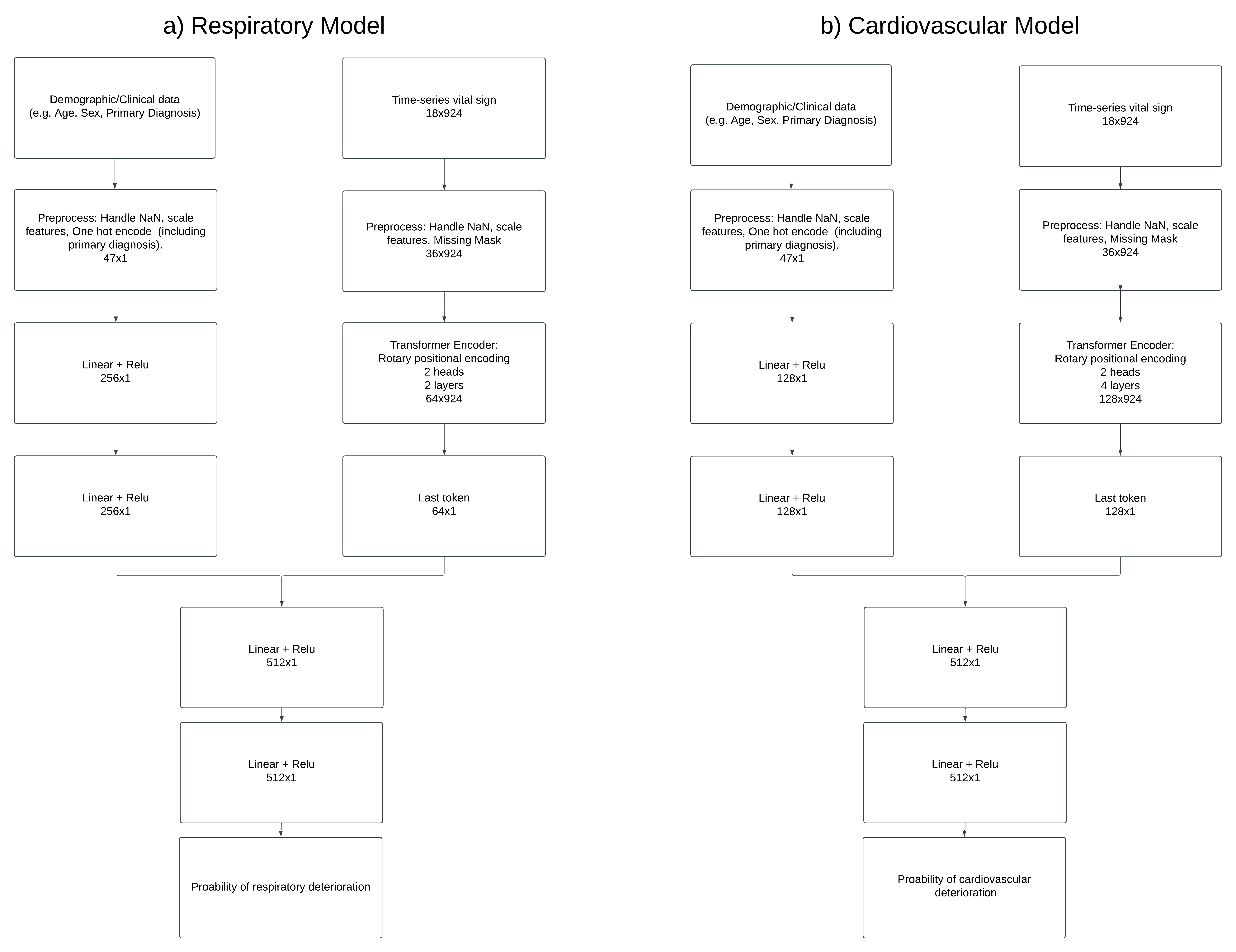


Supplementary Figure 5: Architecture of the Combined Transformer (One-Hot Diagnosis, Reduced Baseline) model. a) Architecture for respiratory model. b) Architecture for cardiovascular model. Each model integrates two branches: a transformer for processing time-series vital signs, and a feed-forward network for a reduced subset of baseline features (including age, weight, sex, PIM3 score, destination care area, pre-existing medical conditions, intra-transport support, and one-hot encoded primary diagnosis). Outputs from both branches are concatenated and passed through a final feed-forward network to generate the prediction.
