# Supplementary material for: Real-time prediction of cardiorespiratory deterioration during paediatric critical care transport using interpretable machine learning
Source: PLOS Digit Health. 2026 May 19;5(5):e0001410. doi: 10.1371/journal.pdig.0001410 (PMC13186380; doi:10.1371/journal.pdig.0001410)
Supplement: S6 Fig — a) Architecture for respiratory model. b) Architecture for cardiovascular model. Each model integrates three branches: a transformer for processing time-series vital signs, a feed-forward network for a reduced subset of baseline features (including age, weight, sex, PIM3 score, destination care area, pre-existing medical conditions, and intra-transport support), and a separate feed-forward network for the vector-embedded primary diagnosis. Outputs from all branches are concatenated and passed through a final feed-forward network to generate the prediction. (DOCX) [file pdig.0001410.s007.docx]

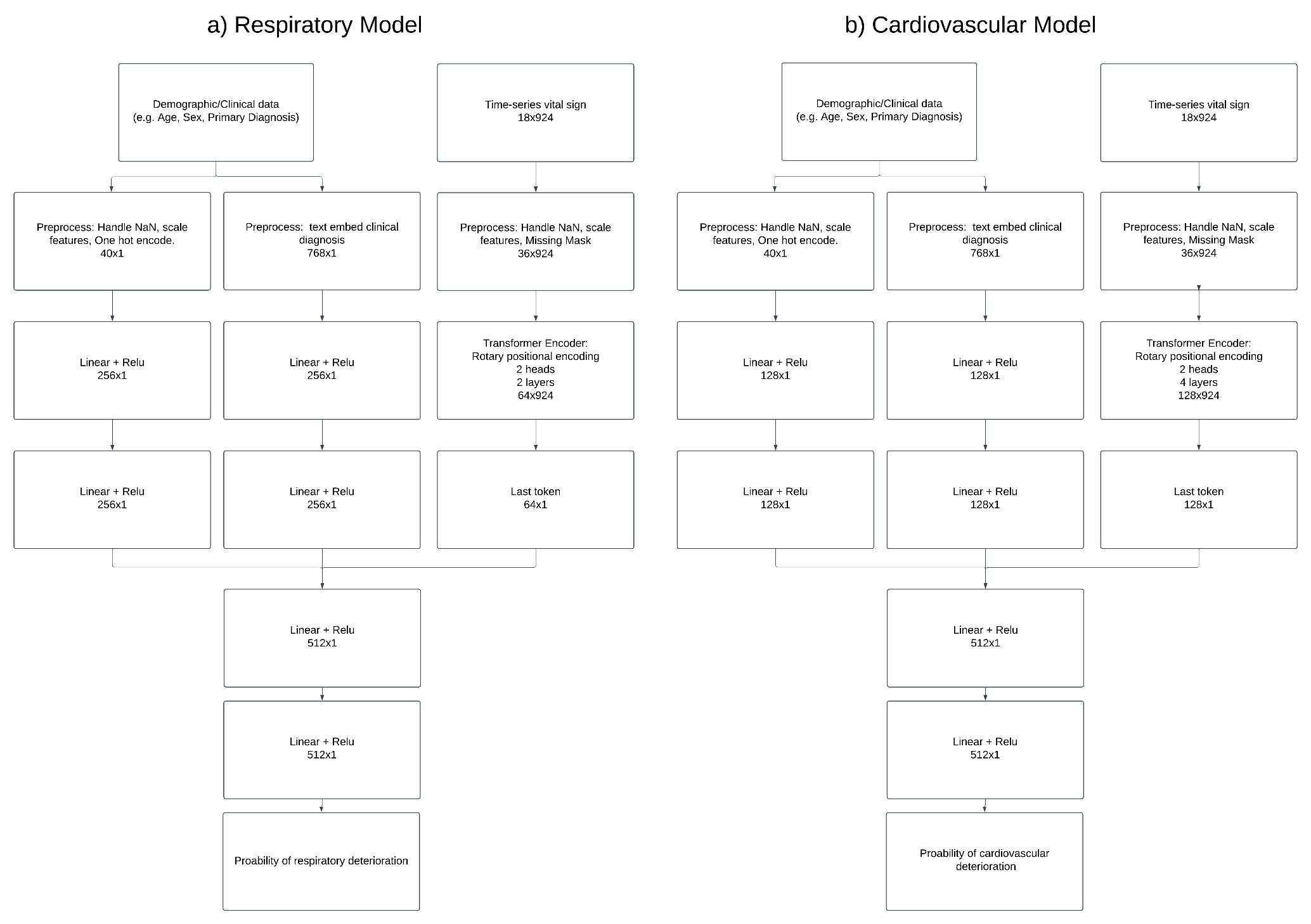


Supplementary Figure 6: Architecture of the Combined Transformer (Vector Diagnosis, Reduced Baseline) model. a) Architecture for respiratory model. b) Architecture for cardiovascular model. Each model integrates three branches: a transformer for processing time-series vital signs, a feed-forward network for a reduced subset of baseline features (including age, weight, sex, PIM3 score, destination care area, pre-existing medical conditions, and intra-transport support), and a separate feed-forward network for the vector-embedded primary diagnosis. Outputs from all branches are concatenated and passed through a final feed-forward network to generate the prediction.
