# Supplementary material for: Real-time prediction of cardiorespiratory deterioration during paediatric critical care transport using interpretable machine learning
Source: PLOS Digit Health. 2026 May 19;5(5):e0001410. doi: 10.1371/journal.pdig.0001410 (PMC13186380; doi:10.1371/journal.pdig.0001410)

Supplementary Figure 8: Study evaluating the impact of historical physiological context. Performance curves for models trained without the 120-minute historical context (utilizing only the immediate 15-minute high-resolution window). (a) Receiver Operating Characteristic (ROC) curve for the respiratory model. (b) Precision-Recall (PR) curve for the respiratory model. (c) ROC curve for the cardiovascular model. (d) PR curve for the cardiovascular model. Comparisons with the primary results (Table 4) demonstrate that excluding the historical context leads to a marked decrease in both discriminative power (AUROC) and precision (AUPRC).


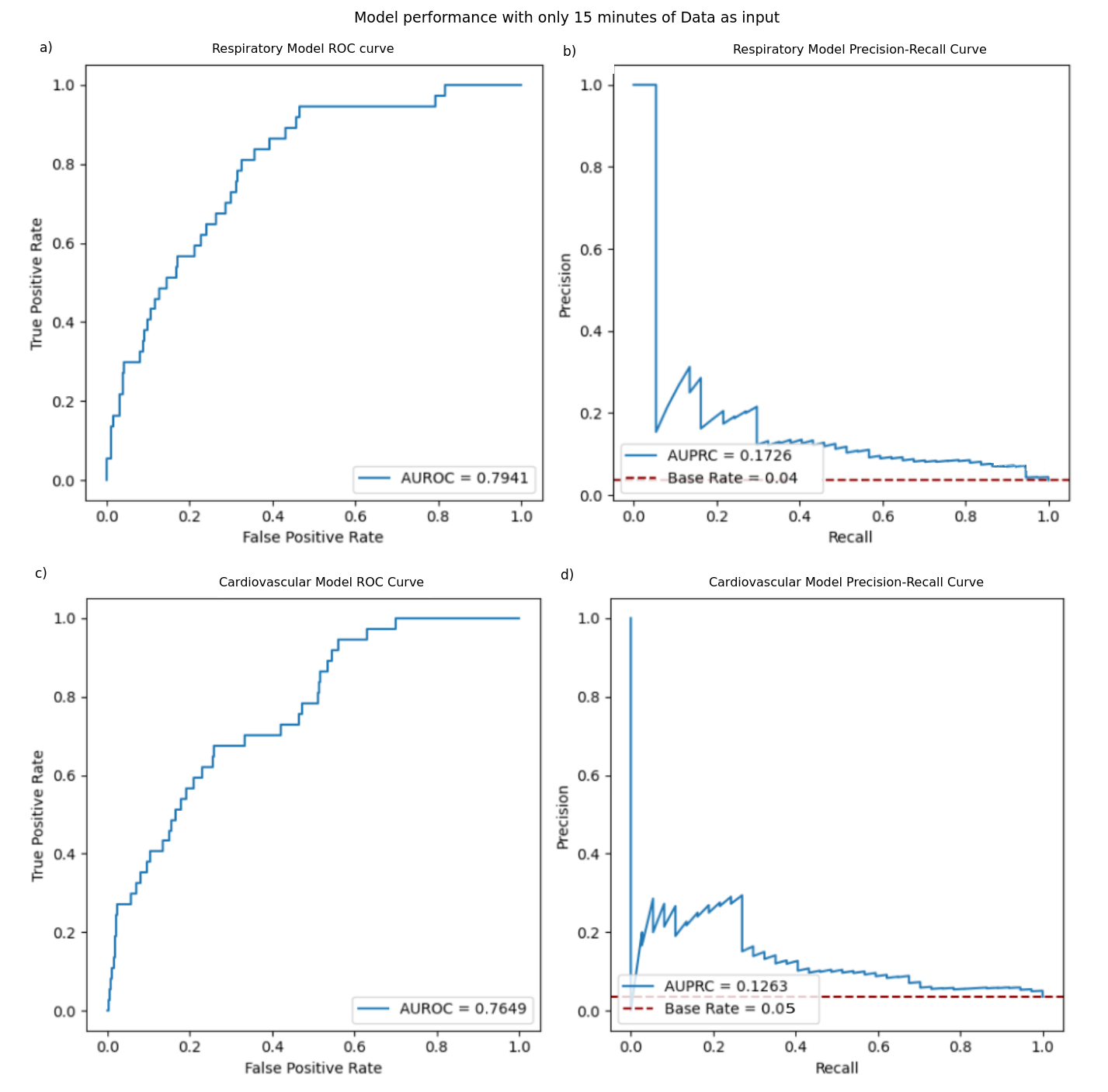

Supplement: S8 Fig — Performance curves for models trained without the 120-minute historical context (utilizing only the immediate 15-minute high-resolution window). (a) Receiver Operating Characteristic (ROC) curve for the respiratory model. (b) Precision-Recall (PR) curve for the respiratory model. (c) ROC curve for the cardiovascular model. (d) PR curve for the cardiovascular model. Comparisons with the primary results (Table 4) demonstrate that excluding the historical context leads to a marked decrease in both discriminative power (AUROC) and precision (AUPRC). (DOCX) [file pdig.0001410.s009.docx]
