# Supplementary material for: Real-time prediction of cardiorespiratory deterioration during paediatric critical care transport using interpretable machine learning
Source: PLOS Digit Health. 2026 May 19;5(5):e0001410. doi: 10.1371/journal.pdig.0001410 (PMC13186380; doi:10.1371/journal.pdig.0001410)
Supplement: Attachment — Submitted filename: Response to Reviewers v0.1.docx [file pdig.0001410.s011.docx]

## **Response to Reviewers: PDIG-D-25-01001**

**Title:** Real-time prediction of cardiorespiratory deterioration during paediatric critical care transport using interpretable machine learning

**Dear PLoS Digital Health team,**

Thank you for the opportunity to revise and resubmit our manuscript, PDIG-D-25-01001, titled "Real-time prediction of cardiorespiratory deterioration during paediatric critical care transport using interpretable machine learning". We appreciate the thorough and constructive feedback from the Section Editor and the reviewers, which has helped us improve our manuscript.

We have addressed all comments raised by the editor and reviewers point-by-point below. Changes in the manuscript are highlighted in the "Revised Manuscript with Track Changes" file.

Reviewer #1

We thank the reviewer for their encouraging comments regarding the clinical relevance of our work and the robustness of the technical approach. We particularly appreciate the detailed feedback regarding the manuscript structure. We agree that the previous organization hindered readability, and we have undertaken a comprehensive restructuring of the manuscript to address these points.

Major Revisions

1. Structure and organization of the manuscript:

- **Reviewer Comment:** “Structure and organization of the manuscript: The current structure impedes a clear understanding of the study design. The "Methods" section is currently presented after the Conclusion. I strongly suggest moving the Methods section (or a comprehensive summary of it) to the main body of the manuscript, before the Results section. Placing the Results before the Methods makes it difficult for the reader to contextualize the findings.

**Response:** We agree that the previous structure made it difficult to contextualize the findings. We have moved the Methods section to the main body of the manuscript, immediately following the Introduction and preceding the Results.

- **Reviewer Comment: “**Mixing Methods with Results: Several concepts presented within the "Results" section actually belong to the methodology.
  For example:
  The sentence "For our predictive analysis, each transport was segmented into 15-minute time windows..." describes data processing, not a result.
  The section titled "Model Performance" largely describes the evaluation setup and metrics used (AUROC, AUPRC, thresholds), which is methodological. Only the reference to Figure 2 and the actual performance outcomes should remain in Results.
  In the "Model Explainability" section, the sentence "We employed the Integrated Gradients method to identify which input features most influenced model predictions" is methodology.”

**Response:** Mixing Methods with Results: We have carefully reviewed the Results section and migrated methodological descriptions to the Methods section. Specifically:

- The explanation of segmenting transports into 15-minute time windows has been moved to the "Window Creation" subsection of Methods.
- The description of evaluation metrics (AUROC, AUPRC, thresholds) has been consolidated in the "Model Evaluation" subsection of Methods.
- The description of the Integrated Gradients technique has been moved to the "Model Explainability" subsection of Methods.
- **Reviewer Comment:** Figure 5 (Study Pipeline): Figure 5 provides a clear overview of the data collection and modeling pipeline. Currently located in the Methods section at the end, this figure should be included in the main body of the manuscript to guide the reader through the study design early on.

**Response:** Study Pipeline Figure: We agree that the study pipeline diagram (previously Figure 5, now Figure 1) provides a crucial overview. We have moved this figure to the beginning of the Methods section to guide the reader through the study design early in the manuscript.

2. Methodology and Data Definitions

- **Reviewer Comment:** Table 2 Clarifications: This table regarding data types and missingness fits better in the Methods section. Furthermore, the definition of ranges is unclear. For example, SpO2 has a range of "0-100". How is a value of 0 interpreted? Is this considered a missing data point or a true physiological value? In a clinical context, a saturation of 0 is a cardiac arrest event, but it is often an artifact or sensor disconnection.

**Response:** We thank the reviewer for this observation. We agree that the data types and missingness table is more appropriately placed in the Methods section and have moved it accordingly (now Table 1).

Regarding the SpO2 range of 0–100, SpO2 values <10 or >100% were filtered out during the 'implausible' filtering stage. We have updated our data preprocessing description to reflect this step.

Manuscript Update: Physiologically implausible values were removed from the	vital sign data	(heart rate <30 or >300 bpm, SpO2 <10 or >100%, EtCO₂ >15	kPa, blood pressure <5 mmHg, temperature <25°C or >45°C).

- **Reviewer Comment:** Ethnicity and Bias: Ethnicity data is missing in over 51% of records. The authors should discuss the potential impact of this high missingness on algorithmic bias and the generalizability of the proposed model.

**Response:** We acknowledge the reviewer’s concern regarding the high rate of missingness in ethnicity data (51.68%). We investigated several model configurations to account for this.

Our results showed that the best-performing models (the "Combined Transformer with Reduced Baseline") achieved optimal AUROC and AUPRC values without the inclusion of ethnicity as an input feature. By excluding ethnicity from the final feature set, we have minimized the risk of the algorithm learning or perpetuating biases based on documented ethnicity.

Furthermore, while the data was missing in the EHR, the physiological signals (high-frequency vital signs) used for prediction are inherently patient-specific. We have added a comment to the Discussion section addressing how the exclusion of ethnicity and the use of individualized physiological baselines helps mitigate bias and improves the generalizability of the model across diverse populations.

Manuscript Updates: Additionally, we observed high rates of missingness in several demographic fields, most notably ethnicity (51.7%). However, our sensitivity analysis revealed that using a reduced feature set (which excluded these sparse demographic variables) not only mitigated potential algorithmic bias but actually improved model performance, likely by reducing noise and allowing the models to focus on high-density physiological signals. This suggests that the immediate physiological trajectory is a more robust predictor of acute deterioration than static baseline characteristics, though it highlights the ongoing challenge of achieving complete data capture in emergency critical care environments.

- **Reviewer Comment:** The missing data rates for Temperature (~70-85%) and Invasive Blood Pressure (>60%) are very high. Please clarify if they were used to train the model or excluded and their impact on bias

Manuscript Update: We thank the reviewer for this important query. Both parameters were included in the model training, but handled differently based on the nature of their missingness:

1. Temperature and Non-Invasive Blood Pressure (NIBP): These parameters are typically recorded intermittently (e.g., every 15 minutes) rather than continuously. To maintain the 1-second resolution required by the Transformer architecture, we utilized a "last observation carried forward" (LOCF) approach. This ensures the model has access to the most recent clinical measurement while acknowledging the lower sampling frequency.

Manuscript Updates: For parameters with lower sampling frequencies	(temperature and Non-Invasive Blood Pressure), a 'last observation carried	forward' (LOCF) approach was used between measurements.

2. Invasive Blood Pressure (IBP): The 63.98% missingness rate for IBP reflects clinical reality: only the most heahaemodynamically unstable patients undergo arterial line insertion. We did not exclude this parameter; instead, we utilized a missingness mask. This allows the model to treat the absence of an arterial line as a feature in itself.

Manuscript Update: For example, the high missingness rate for Invasive Blood	Pressure (63.98%) reflects the proportion of patients without arterial access;	preserving this allowed the model to learn the implicit clinical assessment of	cardiovascular stability associated with the decision not to insert an arterial line.

3. Impact on Bias: In this context, missingness is "informative." The model likely learns that patients without arterial lines are perceived by the clinical team as more cardiovascularly stable. Far from introducing bias, including this "missingness" allows the model to leverage the clinical judgment already exercised by the transport team. Our explainability analysis (Integrated Gradients) confirmed that when IBP was present, it was a high-weight feature for positive predictions, aligning with the clinical expectation that these are higher-risk patients.

Manuscript Update: For example, the high missingness rate for Invasive Blood	Pressure (63.98%) reflects the proportion of patients without arterial access;	preserving this allowed the model to learn the implicit clinical assessment of	cardiovascular stability associated with the decision not to insert an arterial line.

- **Reviewer Comment:** Exclusion Criteria: Please explain the rationale for excluding cases with less than 30 minutes of monitoring data. Does this exclude the most unstable patients who require immediate "scoop and run" transport?

**Response:** We appreciate this opportunity to clarify our exclusion criteria. The 30-minute threshold was chosen for two primary reasons:

1. Model Requirements (Technical): To make a valid 15-minute prediction, the model requires a minimum 15-minute "input window" of high-resolution data to establish a physiological baseline and capture trends. A total duration of less than 30 minutes would not provide sufficient data to generate even a single prediction-label pair (15 minutes input + 15 minutes prediction horizon).
2. Clinical Context of PCCT: Unlike primary emergency "scoop and run" (e.g., roadside to ED), interhospital critical care transports involve a specialist team stabilizing a patient, connecting them to transport monitors, and then transferring them between hospitals. Even in urgent scenarios, the duration from "monitor connection" at the referring site to "handover" at the destination PICU almost always exceeds 30 minutes. Therefore, this criterion is unlikely to have excluded the most unstable patients, but rather filtered out episodes with technical recording failures or extremely short-distance transfers where a predictive tool would have limited actionable utility.

**Manuscript Update:** Given the nature of inter-hospital specialist transport, which includes stabilization and handover phases, 30 minutes represents a minimal clinical duration; thus, this criterion was not expected to exclude high-acuity "scoop and run" cases, but rather to ensure the models had sufficient temporal context for meaningful inference.

- **Reviewer Comment:** The operational definitions of "respiratory deterioration" and "cardiac deterioration" are critical and should be clearly defined in the text before the results are presented.

**Response:** We agree that these definitions are foundational to the study. We have moved the detailed "Label Creation" section within the Methods to ensure the operational definitions of respiratory and cardiovascular deterioration are presented clearly before the Results section.

- **Reveiwer Comment:** Diagnoses: Please clarify the distinction between "Primary diagnosis" and "Existing medical conditions". Does the latter refer specifically to chronic comorbidities?

**Response:** The reviewer is correct. "Primary diagnosis" refers to the acute clinical reason for the paediatric critical care transport (e.g., bronchiolitis, status epilepticus). "Existing medical conditions" refers specifically to the patient’s known chronic co-morbidities (e.g., cerebral palsy, congenital heart disease) present prior to the acute illness. We have updated the "Data Sources" and "Data Processing" sections to reflect this distinction.

Manuscript update: Primary diagnosis was encoded in two ways: via one-hot encoding into clinical categories (respiratory, cardiovascular, neurological, infection, gastroenterology, metabolic, trauma), and via 768-dimensional embeddings from BioClinicalBERT, pre-trained on MIMIC-III.^17^ Embeddings were precomputed and cached in advance to avoid inference-time overhead. Both encoding strategies were tested during model development. Existing co-morbidities were already grouped by organ system and were one-hot encoded.

- **Reviewer Comment**: Diagnoses: Please clarify the distinction between "Primary diagnosis" and "Existing medical conditions". Does the latter refer specifically to chronic comorbidities?

**Response:** To calculate the total transport duration for each episode, we utilized the UTC timestamps associated with the high-frequency vital sign data. Transport time was defined as the temporal difference between the timestamp of the first recorded data point (corresponding to monitor connection at transfer initiation) and the last recorded data point (corresponding to disconnection at patient handover).

Mansucript Update: Methods, Data sources - To calculate the total transport duration for each episode, we utilized the UTC timestamps associated with the high-frequency vital sign data. Transport time was defined as the temporal difference between the timestamp of the first recorded data point (corresponding to monitor connection at transfer initiation) and the last recorded data point (corresponding to disconnection at patient handover).

3. Results

- **Reviewer Comment:** Redundancy: The text in the sections "Respiratory Events" and "Cardiovascular Events" largely repeats the numerical data already presented in Table 4 and Figure 2 . I suggest retaining Table 4 and condensing the text to mention only the most notable findings/trends to improve readability. Perhaps Figure 2 can be placed in supplementary material

**Response:** We agree with the reviewer that the numerical detail was redundant. We have condensed the Results section to focus on the key clinical trends specifically the significant performance boost provided by the transformer-based processing of time-series data and the observation that a reduced baseline feature set outperformed more complex configurations.

- **Reviewer Comment:** Table 4 Placement: This table appears in the manuscript without prior mention in the text.

**Response:** We thank the reviewer for picking up this error and we have ensured that Table 4 is properly cited in the text before the specific results are discussed.

- **Reveriwer Comment:** Table 3 Layout: The layout of Table 3 is confusing; "Total Patients" and "Total Windows" are visually separated from the rest of the data. Additionally, the concepts explained in the table footer regarding class imbalance are important for interpretation and should be included in the main text and not as a foot note

**Response:** We thank the reviewer for this suggestion. We have restructured Table 3 to clearly distinguish between patient-level and window-level frequencies. Furthermore, we have moved the discussion of class imbalance, specifically the observation that while 31% of patients deteriorate, only <10% of time windows are affected, from the table footnote into the primary Results text (under 'Cohort Description'). This change highlights the clinical reality that most of a high-risk transport consists of stable periods, justifying our window-based predictive approach.

Manuscript Update: While approximately 31% of patients experienced at least one form of respiratory or cardiovascular deterioration during their transport, these events were rare relative to the total monitoring time. Specifically, less than 10% of all 15-minute time windows contained a deterioration event. This reflects the high level of class imbalance inherent in transport data; even patients who eventually deteriorate remain physiologically stable for the vast majority of the transfer duration.

Minor revisions:

- **Reviewer Comment:** Bibliography: The references do not currently follow the Vancouver format required by the journal.

**Response:** We have addressed the formatting concerns by restructuring the Bibliography into the Vancouver format as required by the journal.

- **Reviewer Comment:** Glossary: It would be appropriate to include a glossary or figure legend defining the metrics used (AUROC, AUPRC, F1-score) to assist clinical readers in the comprehension of the manuscript

**Response:** We have addressed the request for a glossary by expanding the legend of Table 4. This legend now defines the clinical and statistical implications of AUROC, AUPRC, PPV, and F1-score, ensuring that clinical readers can interpret the performance results without referring to external definitions.

Reviewer #2

1. **Revewier Comment:** The authors used Integrated Gradients method for model explaination, but cohort-level feature importance based on absolute attributions can obscure directionality (protective vs risky). Please include individual examples showing feature importance over time and propose how explanations would be displayed to clinicians in real-time.

**Response:** Integrated Gradients: We thank the reviewer for this insightful comment. We agree that for an AI model to be clinically useful in a high-stakes environment like paediatric critical care transport, it must move beyond "black-box" predictions and provide actionable insights. We have addressed this by adding a new figure (Figure 7) and expanding the Discussion section to propose a framework for real-time explainable alerting.

a. Visual Evidence of Clinical Synchronization (Figure 7): We have added a new Figure 7 which demonstrates the model’s responsiveness to specific clinical events in a 2-year-old patient. This figure shows that the model’s "risk attribution" (calculated via Integrated Gradients) spikes in direct temporal alignment with localized desaturation events and hypertensive spikes. This provides visual evidence that the model’s internal logic is well-synchronized with established paediatric clinical thresholds, which is a prerequisite for clinician trust.
b. Proposed Clinical Interface (Discussion): We have amended the Discussion of explainability to describe how these explanations could be displayed to transport teams. Rather than a solitary probability score, we propose an interface that flags the specific physiological parameters driving a "High Risk" prediction. For example, a clinician would see a "Respiratory Risk" alert accompanied by a notification that the risk is primarily driven by "instabilities in EtCO2 and SpO2."

Manscript Update: From a clinical interface perspective, these feature attributions could be translated into real-time "explainable alerts" on transport monitors. Rather than presenting a solitary risk probability, the system could dynamically flag the top-contributing physiological parameters; for example, explicitly indicating that a rising respiratory risk score is being driven by specific instabilities in EtCO2 and SpO2. Such an approach allows clinicians to rapidly cross-reference the AI’s logic with their own clinical assessment, facilitating more targeted interventions and reducing the cognitive load associated with interpreting raw high-frequency data in a high-pressure environment.

2. **Reveiwer Comment:** While AUROCs are good, but the AUPRCs, PPVs are not high, largely due to low event prevalence. The authors discuss alarm fatigue, but further clarification is needed on how clinicians should interpret risk outputs in practice. For example, what magnitude of predicted probability would justify clinical intervention? How many false alerts per hour would be acceptable operationally? Providing a clearer clinical integration pathway would improve translational clarity.

**Response:** AUROC/AUPCR/ Alarm fatigue: We thank the reviewer for this critical point regarding the translational pathway. We acknowledge that the low prevalence of events (approx. 4–5% of windows) results in modest PPVs, a common challenge in predicting rare acute events.

a. Adjunctive Decision Support: We emphasize that these models are intended as adjunctive tools rather than autonomous diagnostic systems. The AI is designed to "nudge" the clinician’s attention toward evolving patterns that may be subtle, not to mandate a specific intervention.
b. Context-Dependent Thresholding: We agree that a "one-size-fits-all" probability threshold is inappropriate for a diverse paediatric population. The optimal balance between sensitivity (catching every event) and specificity (minimizing alarm fatigue) is highly context-dependent, varying by clinician experience, patient acuity, and the specific transport environment.
c. Operational Demonstration: For the purposes of this study, we demonstrated a threshold based on a 20% False Positive Rate (FPR) to provide a performance baseline. However, in practice, we envision a system where alert sensitivity could be calibrated by the user or the unit’s operational protocols. We have expanded the Discussion to clarify this clinical integration pathway.

Manuscript Update: Crucially, we do not envision these models as autonomous diagnostic tools, but rather as adjunctive digital safety nets designed to complement, not replace, clinical judgment. The specific probability threshold that should justify intervention is not a "one-size-fits-all" value; rather, it is highly context-dependent and likely to vary based on regional protocols, patient acuity, and the clinician’s experience. By providing a customizable and explainable output, the model allows practitioners to calibrate the "digital nudge" to their specific operational tolerance

3. **Reviewer Comment:** The manuscript frequently describes the proposed models as “lightweight,” but this claim would benefit from clearer quantitative justification. I recommend providing objective evidence such as parameter counts, memory footprint, inference latency, and computational requirements on representative edge hardware to substantiate the lightweight deployment claim.

**Response:** Model Lightweight quantification: We thank the reviewer for this suggestion of providing objective evidence such as parameter counts, memory footprint, inference latency, and computational requirements. We have added a dedicated section to the Results and Methods to quantify the models' "lightweight" status.

We conducted these benchmarks locally on a consumer grade laptop (Intel Core i7) without any remote server or GPU assistance, specifically to simulate the processing power available in a typical transport setting.

Manuscript Update: Computational Efficiency: Both top-performing models demonstrated high computational efficiency suitable for edge deployment. The parameter counts for the respiratory and cardiovascular models were <700,000, with a serialized memory footprint of <2.7 MB per model. When benchmarked locally on a standard laptop CPU, the average inference latency was <60 ms per 15-minute data window.
4. **Reviewer Comment:** The authors emphasize edge-device compatibility but lacks details on implementation challenges in actual transport environments. For example: How would missing data be handled in real time? What is the fallback mechanism if the model fails to generate a prediction?

**Response:** Implementation challenges: We thank the reviewer for highlighting these practical safety considerations. We have addressed these implementation challenges through both the model design and the proposed clinical workflow:

A. Handling Missing Data: As detailed in the Methods (Data Processing), the models were specifically trained on real-world transport data which included frequent signal dropouts and "informative missingness." The use of a missingness mask allows the model to maintain inference even when specific parameters (e.g., invasive blood pressure) are unavailable. In a real-time environment, the model continues to provide a risk estimate based on the remaining available features, mirroring the clinician’s own need to make decisions with incomplete data.
B. Offline Reliability: By designing the models to run locally (offline) on edge devices, we have eliminated the primary failure mode of remote AI systems: connectivity loss. This "offline-first" approach ensures that model failure is limited only to hardware malfunction.
C. Fallback Mechanism (Adjunctive Use): We reiterate that these models are adjunctive tools. The "fallback" is the standard of care; the continuous monitoring of raw vital signs and the clinician’s expert judgment. If the model fails to generate a prediction (e.g., due to a software crash), the clinician is not "blinded," as the primary physiological monitor remains the gold standard.

Manuscript Update: Crucially, we do not envision these models as autonomous diagnostic tools, but rather as adjunctive digital safety nets designed to complement, not replace, clinical judgment. The specific probability threshold that should justify intervention is not a "one-size-fits-all" value; rather, it is highly context-dependent and likely to vary based on regional protocols, patient acuity, and the clinician’s experience. By providing a customizable and explainable output, the model allows practitioners to calibrate the "digital nudge" to their specific operational tolerance

Reviewer 3

1. **Revewier Comment:** The manuscript lacked details on the model architecture. I was not clear on what each variant was tested until I read the supplements. The figures depicting the model architecture was confusing as well, especially around vital signs. For example, how was the 18x924 shape was obtained, and subsequently how does preprocessing lead to 36x924

**Response:** We thank the reviewer for the opportunity to clarify the architectural details and the dimensionality of our input tensors.

a. Derivation of the 924x18 Shape: The input sequence is constructed by concatenating two distinct temporal contexts. We utilize 24 data points representing 5-minute averages to provide up to 120 minutes of historical trajectory, followed by 900 data points of high-frequency (1 Hz) vital signs from the immediate 15-minute prediction window. This results in a unified input sequence length of 924 tokens. The 18 features consist of the 16 physiological parameters (Table 1) and 2 binary indicators for previously occurring adverse events, ensuring the model maintains both long-term clinical trends and acute physiological volatility
b. Expansion to 924x36 (Dual-Channel Encoding): The transition from 18 to 36 features represents the concatenation of the standardized physiological values with a corresponding binary "Informative Missingness Mask." For every input feature, a binary indicator (1 = present, 0 = missing) is generated. These two 924x18 matrices are concatenated along the feature dimension. This approach ensures that the Transformer’s self-attention mechanism can explicitly distinguish between true physiological signals and mean-imputed values during sensor dropouts.

Manscript Update: To enable the model to distinguish between observed and imputed data, a binary missingness mask (1 = observed, 0 = imputed) was generated for each of the 18 input features. This mask was concatenated with the physiological values along the feature dimension, resulting in a dual-channel input vector of 36 features per time step.

2. **Reviewer Comment:** Connected to point 1, how were the vitals input processed for the transformer blocks? The methods section also lacked details on that.

**Response:** We thank the reviewer for the opportunity to clarify our vital signal processing pipeline. We have updated the Methods section (Data Processing and Model Development subsections) to more explicitly describe how the input tensors are structured to ensure numerical stability and temporal awareness:

a. Normalization: All vital signs were z-score normalized or scaled (as detailed in the *Data Processing* section) prior to model input. This ensures that all 18 features occupy a comparable numerical range, preventing features with larger absolute magnitudes from disproportionately influencing the gradient updates.

Manuscript Update: Physiologically implausible values were removed from the vital sign data (heart rate <30 or >300 bpm, SpO2 <10 or >100%, EtCO₂ >15 kPa, blood pressure <5 mmHg, temperature <25°C or >45°C). To account for age-related variability, all vital signs were standardised to improve comparability and enhance the reliability of model training. Respiratory rate, heart rate, and blood pressure were z-score normalised following methodology outlined in our prior work.^5^ SpO₂ was scaled using (SpO₂ − 97)/6, ETCO₂ using (ETCO₂ − 5.25)/1.5, and temperature using (Temp − 36.75)/1.5.

b. Temporal Encoding: To preserve the sequence of events without the constraints of absolute positional encodings, we utilized Rotary Positional Embeddings (RoPE) within the Transformer blocks. RoPE allows the model to leverage the relative distance between physiological shifts. This is particularly valuable for our architecture, as it enables the model to effectively integrate trends across both the 120-minute historical context and the 15-minute high-resolution acute window.

Manuscript Update: To optimize temporal encoding, transformer blocks were implemented with rotary positional embeddings.^20^ Transformer blocks were implemented with a decoder-only architecture to ensure data points could only attend to preceding data, thereby guaranteeing that predictions were based solely on historical information.^19^ Given the class imbalance (adverse events being rare) we applied class-weighted loss during training to penalize false negatives more heavily to maintain balanced performance across classes.

3. **Reveiwer Comment:** While I appreciate that the authors are transparent with the amount of missing data and the small dataset size, the lack of data does show up in the results in terms of how jagged the curves are Figure 2. It was not clear how the confidence interval was calculated also.

**Response:** Curve shape and confidence intervals:

a. Confidence Intervals: We apologize for the lack of clarity in the initial draft. The 95% confidence intervals (CIs) for all performance metrics were calculated using a stratified bootstrapping approach on the holdout test set with 5,000 iterations. We have updated our Methods (Model evaluation) to explain this clearly.

Manuscript Updates: To provide a robust estimate of uncertainty and ensure the reliability of our findings, 95% confidence intervals (CIs) for all performance metrics were calculated using a stratified bootstrapping approach on the test set with 5,000 iterations.

b. Dataset Size and Curve Morphology: We acknowledge the limited size of the dataset and its impact on the morphology of the ROC and PR curves. While we agree that a larger dataset would be ideal for achieving smoother curves and potentially higher precision, we are utilizing a real-world, clinical dataset captured in the highly challenging environment of critical care transport. We believe that reporting results from this authentic clinical cohort (despite its size) is essential for demonstrating the feasibility of the approach. We have noted in the Discussion that expanding the data volume to further refine and validate these models is a primary objective for future multi-centre research.

Manuscript Update: Looking ahead, external validation is essential before real-world deployment. Testing on datasets from other paediatric transport services (both nationally and internationally) will be crucial to assess generalisability across different patient populations, transport systems, monitoring technologies, and clinical practices. Multicentre validation would also enable retraining or fine-tuning on more heterogeneous data, improving robustness and broader applicability. Following this, prospective deployment studies will be needed to evaluate real-world performance, clinical decision-making and ultimately, patient outcomes.

4. **Reveiwer Comment:** The authors mentioned the operational constraint that only two recording devices are available and technical issues limited the amount of data available. Is that the case across health systems? As the models hedges on data being available, those sound like big hurdles to overcome even for model validation and deployments. I am also concerned with how such issues can create bias in the data and how well the results translate to a larger set of patients. The authors should elaborate and address these concerns in the discussion.

**Response:** We thank the reviewer for highlighting these operational challenges. We would like to clarify the nature of this constraint and have revised the limitations section of our discussion accordingly:

A. Universal Monitoring: All patients transported by the CATS team receive the same high standard of continuous physiological monitoring. The "two-device" constraint was purely a research data-capture limitation. Only two of the three active transport teams were equipped with the hardware necessary to transmit these high-frequency data to a secure remote research server.
B. Lack of Selection Bias: The assignment of these specific devices was based on ambulance rotation and team availability, not on patient acuity or diagnosis. As detailed in the Results (Table 2), we conducted a detailed comparison between the included cohort and the total transport population. The included patients were demographically and clinically representative across all major categories, which significantly minimizes the risk of selection bias.
C. Data Availability: We agree that data availability is a hurdle. However, we believe that research such as ours, demonstrating the high predictive value of stored physiological data, is a key driver for securing future investment in data-capture systems. By validating the model on this representative subset, we provide the necessary proof-of-concept to justify broader institutional and regional deployment of data-storage infrastructure.

Manuscript Update: It is important to clarify that the limited data capture (23.7% of transports) was a result of research-specific logistical constraints rather than clinical selection; while all patients were continuously monitored, only two of three transport teams were equipped with the hardware necessary to transmit high-frequency data to a secure research server. Because device assignment was determined by vehicle rotation and hardware availability rather than patient acuity, the risk of selection bias was minimized. This is substantiated by our analysis in Table 2, which demonstrates that the included cohort remained demographically and clinically representative of the total transport population (n=6,471) across age, diagnosis, and illness severity. Furthermore, while these data-capture hurdles are significant across health systems, proof-of-concept studies like this are essential to demonstrate the high clinical utility of physiological data storage, advocating for the future expansion of data infrastructure.

5. **Reveiwer Comment:** How much does vitals outside the 15 min window contribute? It would be interesting to see another variant using only the 15 min vitals.

**Response:** Vitals outside the 15 minutes: We thank the reviewer for this suggestion. As part of our model development and optimization phase, we conducted an ablation study specifically to quantify the predictive gain provided by the 120-minute historical context.

We compared our best-performing Combined Transformer models against variants trained and tested using only the immediate 15-minute high-resolution vital sign window. We have now integrated these findings into our Results section and provided the corresponding ROC and Precision-Recall curves in a new Supplementary Figure 8.

The results demonstrate that the inclusion of historical context was a key factor in improving performance:

- Respiratory Model: AUROC increased from 0.794 to 0.851; AUPRC increased from 0.173 to 0.200.
- Cardiovascular Model: AUROC increased from 0.765 to 0.792; AUPRC increased from 0.126 to 0.183.

This confirms that the long-term physiological trajectory provides essential context for the Transformer to accurately interpret acute changes in patient state.

Manuscript Update: Impact of Temporal Context: To evaluate the contribution of the upto 120-minute historical context, we compared the top-performing Combined Transformer models against baseline variants trained on only immediate 15-minute input window. The inclusion of the two-hour historical trajectory resulted in a substantial performance boost across both tasks. For respiratory deterioration, the AUROC improved from 0.794 to 0.851 and the AUPRC from 0.173 to 0.200. Similarly, cardiovascular predictive performance rose from an AUROC of 0.765 to 0.792 and an AUPRC of 0.126 to 0.183 (Supplementary Figure 8). These results indicate that the patient’s prior physiological trend provides essential context that allows the models to more accurately interpret acute shifts in vital signs.

6. **Reveiwer Comment:** As the models predict events, how would such predictions lead to actions? Discussion should be expanded with how clinicians could benefit from this tool.

**Response:** We thank the reviewer for this prompt to bridge the gap between predictive accuracy and clinical action. While we initially framed the model as a "digital safety net," we have now expanded the Discussion to detail specific clinical scenarios where a 15-minute lead time provides a critical window for intervention.

Manuscript Updates: From a clinical interface perspective, these feature attributions could be translated into real-time "explainable alerts" on transport monitors. Rather than presenting a solitary risk probability, the system could dynamically flag the top-contributing physiological parameters; for example, explicitly indicating that a rising respiratory risk score is being driven by specific instabilities in EtCO2 and SpO2. Such an approach allows clinicians to rapidly cross-reference the AI’s logic with their own clinical assessment, facilitating more targeted interventions and reducing the cognitive load associated with interpreting raw high-frequency data in a high-pressure environment. By transforming this high-frequency data into an actionable 15-minute lead time, the model enables a shift from reactive crisis management to proactive stabilization. For example, a rising respiratory risk attribution could prompt a clinician to perform a focused assessment of the endotracheal tube position or optimize recruitment manoeuvrers before a significant desaturation occurs, ultimately facilitating smoother, safer transitions of care.

Minor Revisions

1. **Reveiwer Comment:** Figures are in poor quality. The AUROC/PR curves are very pixelated.

**Response:**	We apologize for the degradation in image quality; the pixelation was an artifact of the image compression during insertion into the Word manuscript file. High resolution files have been uploaded separately to the submission system.

2. **Reveiwer Comment:** There’s a reference error in Supplementary Table 1. I am seeing Error! Reference source not found under the Input Features column.

**Response:** We thank the reviewer for spotting this. The broken hyperlink has now been corrected.

3. **Reveiwer Comment:** The bolding in Table 4 is confusing. The AUROC bolded is not the best model.

**Response:** We appreciate the reviewer in noting this error. We have corrected the bolding to highlight the top performing model.

4. **Reveiwer Comment:** It is not clear what the feature set is, or what is being reduced in the reduced variants.

**Response:** We appreciate the reviewer for highlighting this lack of clarity. We have updated the Methods to provide explicit definitions. The Full Baseline includes 30 variables (all EHR data, including sparse demographic fields pre-transport interventions). The Reduced Baseline was restricted to 9 core clinical variables (Age, Weight, Sex, PIM3, Destination Area, Diagnosis, Pre-existing conditions, and Respiratory/Cardiovascular support).

Mansuscript updates: To evaluate the minimal configuration required for real-time deployment, we compared two baseline feature sets. The ‘Full Baseline’ configuration utilized 30 variables from the EHR, including all demographic data and the exhaustive list of pre-transport clinical interventions shown in Table 1. The ‘Reduced Baseline’ configuration was restricted to 9 core clinical variables: age, weight, sex, PIM3 score, destination care area, primary diagnosis, pre-existing medical conditions, and intra-transport respiratory and cardiovascular support. This reduction intentionally excluded sparse or inconsistently recorded demographic fields and was intended to minimise the manual data entry burden for clinicians during high-acuity transport, ensuring that the model requires only the nine most critical clinical variables for immediate inference.
