# Supplementary material for: Real-time prediction of cardiorespiratory deterioration during paediatric critical care transport using interpretable machine learning
Source: PLOS Digit Health. 2026 May 19;5(5):e0001410. doi: 10.1371/journal.pdig.0001410 (PMC13186380; doi:10.1371/journal.pdig.0001410)
Supplement: Attachment — Submitted filename: Response to Reviewers v0.2.docx [file pdig.0001410.s012.docx]

## Response to Reviewers: PDIG-D-25-01001

Title: Real-time prediction of cardiorespiratory deterioration during paediatric critical care transport using interpretable machine learning

Dear PLoS Digital Health team,

Thank you for the opportunity to revise and resubmit our manuscript, PDIG-D-25-01001, titled "Real-time prediction of cardiorespiratory deterioration during paediatric critical care transport using interpretable machine learning". We appreciate the thorough and constructive feedback from the Section Editor and the reviewers, which has helped us improve our manuscript.

We have addressed all comments raised by the editor and reviewers point-by-point below. Changes in the manuscript are highlighted in the "Revised Manuscript with Track Changes" file.

Reviewer #1

1. **Reviewer Comment:** Evaluation of Bias and Fairness: regarding the handling of demographic variables and potential bias: it is important to emphasize that excluding sensitive variables is not necessarily the most effective way to mitigate bias. Machine learning models frequently learn from implicit biases already embedded in clinical data (such as practice patterns or systemic inequities). Therefore, a more robust way to evaluate and address bias is to explicitly analyze and report the model's performance across different subgroups, for example patients with missing race-etnicity. This ensures that the predictive accuracy remains consistent regardless of the patient's demographic background, rather than assuming that removing the variable eliminates the underlying bias.

**Response:** We thank the reviewer for this critical insight. We agree that the exclusion of sensitive attributes is not a definitive safeguard against algorithmic bias, as models can inadvertently leverage clinical practice patterns as proxies for systemic inequities.

We carefully reviewed the ethnicity distribution within our holdout test set (n=155). We found that 51% (n=79) of the test set had unknown or missing ethnicity data. Among the remaining patients, the distribution was highly fragmented across 12 distinct ethnic categories, resulting in several groups being represented by only a single patient (e.g., n=1 for Asian Bangladeshi and n=1 for Mixed White and Black Caribbean).

Given that the clinical deterioration events we are predicting are themselves rare (occurring in <5% of time windows), evaluating performance on such small subgroups would lack the statistical power to provide reliable conclusions. Calculating metrics like AUROC or Sensitivity on a sample size of n=1 would yield unstable and potentially misleading results that do not accurately reflect the model’s true performance or equity.

We have significantly revised the Discussion to address this point. We now explicitly acknowledge the limitations of variable exclusion and frame this study as a physiological proof-of-concept. We emphasize that a mandatory requirement for future prospective validation on a larger and more diverse cohort will be a powered, pre-specified subgroup analysis to ensure the model performs equitably across all demographic profiles, including those with missing data

**Manuscript updates:** Discussion: Additionally, we observed high rates of missingness in several demographic fields, most notably ethnicity (51.7%). While our sensitivity analysis revealed that a reduced feature set (excluding these sparse demographic variables) improved model performance by focusing on high-density physiological signals, we acknowledge that the exclusion of sensitive attributes does not fully insulate a model from bias. Clinical data often contains embedded systemic inequities that machine learning can inadvertently learn. In this retrospective study, a robust subgroup analysis for fairness was not statistically viable due to the highly fragmented distribution of ethnicity in the holdout test set (n=155), where 79 patients had unknown ethnicity and several minority categories were represented by only a single patient. Evaluating performance on such small subgroups would yield unstable and potentially misleading results.These findings suggest that while the immediate physiological trajectory is a more robust predictor of acute deterioration than static baseline characteristics, achieving complete data capture in emergency environments remains an ongoing challenge. Proof-of-concept studies like this are essential to advocate for the future expansion of clinical data infrastructure and larger, more diverse cohorts where powered, pre-specified subgroup analyses can appropriately evaluate model performance across all demographic backgrounds.

2. **Reviewer Comment:** Figure References: in the "Data Sources" section, specifically at line 8, you mention a visual representation of the data, but the specific figure number is missing. Please ensure this reference is completed to maintain the manuscript's technical clarity.

**Response:** We thank the reviewer for highlighting this error. We have updated the manscript accordingly as follows “Data collection starts at transfer initiation, with ambulance staff using a SwiftCare-enabled smartphone to connect wirelessly to a Philips Intellivue MP5 monitor, recording continuously until patient handover at the destination unit (Figure 1).”

3. **Reviewer Comment:**  I noticed that specific tables are still being cited within the Discussion section. The Discussion should focus on the conceptual interpretation and clinical implications of your findings. I suggest avoiding direct citations of tables in this section to allow for a more fluid narrative, as the primary data has already been presented and referenced in the Results section.

**Response:** We thank the reviewer for noticing this point. We have removed redundant references within the discussion section to improve flow.
