# Supplementary material for: Real-time prediction of cardiorespiratory deterioration during paediatric critical care transport using interpretable machine learning
Source: PLOS Digit Health. 2026 May 19;5(5):e0001410. doi: 10.1371/journal.pdig.0001410 (PMC13186380; doi:10.1371/journal.pdig.0001410)
Supplement: Peer review history

# Peer review: Decision Letter 0

**DOI:** 10.1371/journal.pdig.0001410.r001

**Authors:** Chenxi Yang

15 Jan 2026

PDIG-D-25-01001

Real-time prediction of cardiorespiratory deterioration during paediatric critical care transport using interpretable machine learning

PLOS Digital Health

Dear Dr. Kapur,

Thank you for submitting your manuscript to PLOS Digital Health. After careful consideration, we feel that it has merit but does not fully meet PLOS Digital Health's publication criteria as it currently stands. Therefore, we invite you to submit a revised version of the manuscript that addresses the points raised during the review process.

Please submit your revised manuscript by Mar 16 2026 11:59PM. If you will need more time than this to complete your revisions, please reply to this message or contact the journal office at digitalhealth@plos.org. When you're ready to submit your revision, log on to https://www.editorialmanager.com/pdig/ and select the 'Submissions Needing Revision' folder to locate your manuscript file.

Please include the following items when submitting your revised manuscript:

* A letter that responds to each point raised by the editor and reviewer(s). You should upload this letter as a separate file labeled 'Response to Reviewers'. This file does not need to include responses to any formatting updates and technical items listed in the 'Journal Requirements' section below.

* A marked-up copy of your manuscript that highlights changes made to the original version. You should upload this as a separate file labeled 'Revised Manuscript with Track Changes'.

* An unmarked version of your revised paper without tracked changes. You should upload this as a separate file labeled 'Manuscript'.

If you would like to make changes to your financial disclosure, competing interests statement, or data availability statement, please make these updates within the submission form at the time of resubmission. Guidelines for resubmitting your figure files are available below the reviewer comments at the end of this letter.

We look forward to receiving your revised manuscript.

Kind regards,

Chenxi Yang, Ph.D.

Academic Editor

PLOS Digital Health

Chenxi Yang

Academic Editor

PLOS Digital Health

Leo Anthony Celi

Editor-in-Chief

PLOS Digital Health

orcid.org/0000-0001-6712-6626

**Journal Requirements:**

If the reviewer comments include a recommendation to cite specific previously published works, please review and evaluate these publications to determine whether they are relevant and should be cited. There is no requirement to cite these works unless the editor has indicated otherwise.

**Additional Editor Comments (if provided):**

[Note: HTML markup is below. Please do not edit.]

**Reviewers' Comments:**

Reviewer's Responses to Questions

**Comments to the Author**

1. Does this manuscript meet PLOS Digital Health’s publication criteria? Is the manuscript technically sound, and do the data support the conclusions? The manuscript must describe methodologically and ethically rigorous research with conclusions that are appropriately drawn based on the data presented.

Reviewer #1: Yes

Reviewer #2: Partly

Reviewer #3: Partly

**********

2. Has the statistical analysis been performed appropriately and rigorously?

Reviewer #1: Yes

Reviewer #2: Yes

Reviewer #3: Yes

**********

3. Have the authors made all data underlying the findings in their manuscript fully available (please refer to the Data Availability Statement at the start of the manuscript PDF file)?

The PLOS Data policy requires authors to make all data underlying the findings described in their manuscript fully available without restriction, with rare exception. The data should be provided as part of the manuscript or its supporting information, or deposited to a public repository. For example, in addition to summary statistics, the data points behind means, medians and variance measures should be available. If there are restrictions on publicly sharing data—e.g. participant privacy or use of data from a third party—those must be specified.

Reviewer #1: Yes

Reviewer #2: Yes

Reviewer #3: No

**********

4. Is the manuscript presented in an intelligible fashion and written in standard English?

PLOS Digital Health does not copyedit accepted manuscripts, so the language in submitted articles must be clear, correct, and unambiguous. Any typographical or grammatical errors should be corrected at revision, so please note any specific errors here.

Reviewer #1: No

Reviewer #2: Yes

Reviewer #3: Yes

**********

5. Review Comments to the Author

Please use the space provided to explain your answers to the questions above. You may also include additional comments for the author, including concerns about dual publication, research ethics, or publication ethics. (Please upload your review as an attachment if it exceeds 20,000 characters)

Reviewer #1: I read this manuscript with great interest and I would like to congratulate the authors for addressing such a critical topic in pediatric intensive care. The inter-hospital transport of critically ill children inherently exposes patients to life-threatening risks; therefore, the ability to predict physiological instability in advance is of high clinical relevance and is necessary to enhance patient safety.

While the subject matter is significant and the technical approach appears robust, the manuscript requires restructuring prior to publication to facilitate reader comprehension. Currently, the organization of the sections blurs the line between methodology and results, and the presentation of data needs further clarification to ensure reproducibility and clinical interpretability. Below are specific comments organized by section and importance.

I expect my suggestions help to enhance the manuscript for readers who are not experts in the subject

Major Revisions

1. Structure and organization of the manuscript: The current structure impedes a clear understanding of the study design. The "Methods" section is currently presented after the Conclusion. I strongly suggest moving the Methods section (or a comprehensive summary of it) to the main body of the manuscript, before the Results section. Placing the Results before the Methods makes it difficult for the reader to contextualize the findings.

Mixing Methods with Results: Several concepts presented within the "Results" section actually belong to the methodology.

For example:

The sentence "For our predictive analysis, each transport was segmented into 15-minute time windows..." describes data processing, not a result.

The section titled "Model Performance" largely describes the evaluation setup and metrics used (AUROC, AUPRC, thresholds), which is methodological. Only the reference to Figure 2 and the actual performance outcomes should remain in Results.

In the "Model Explainability" section, the sentence "We employed the Integrated Gradients method to identify which input features most influenced model predictions" is methodology.

Figure 5 (Study Pipeline): Figure 5 provides a clear overview of the data collection and modeling pipeline. Currently located in the Methods section at the end, this figure should be included in the main body of the manuscript to guide the reader through the study design early on.

2. Methodology and Data Definitions

Table 2 Clarifications: This table regarding data types and missingness fits better in the Methods section. Furthermore, the definition of ranges is unclear. For example, SpO2 has a range of "0-100". How is a value of 0 interpreted? Is this considered a missing data point or a true physiological value? In a clinical context, a saturation of 0 is a cardiac arrest event, but it is often an artifact or sensor disconnection.

Ethnicity and Bias: Ethnicity data is missing in over 51% of records. The authors should discuss the potential impact of this high missingness on algorithmic bias and the generalizability of the proposed model.

Vital Signs Missingness: The missing data rates for Temperature (~70-85%) and Invasive Blood Pressure (>60%) are very high. Please clarify if they were used to train the model or excluded and their impact on bias

Exclusion Criteria: Please explain the rationale for excluding cases with less than 30 minutes of monitoring data. Does this exclude the most unstable patients who require immediate "scoop and run" transport?

Operational Definitions:

The operational definitions of "respiratory deterioration" and "cardiac deterioration" are critical and should be clearly defined in the text before the results are presented.

Diagnoses: Please clarify the distinction between "Primary diagnosis" and "Existing medical conditions". Does the latter refer specifically to chronic comorbidities?

Transport Time: Please include how "Overall transport time" was calculated in the main text of the manuscript

3. Presentation of Results

Redundancy: The text in the sections "Respiratory Events" and "Cardiovascular Events" largely repeats the numerical data already presented in Table 4 and Figure 2 . I suggest retaining Table 4 and condensing the text to mention only the most notable findings/trends to improve readability. Perhaps Figure 2 can be placed in supplementary material

Table 4 Placement: This table appears in the manuscript without prior mention in the text.

Table 3 Layout: The layout of Table 3 is confusing; "Total Patients" and "Total Windows" are visually separated from the rest of the data. Additionally, the concepts explained in the table footer regarding class imbalance are important for interpretation and should be included in the main text and not as a foot note

Figure 2 Interpretation: While the text only refers to Figure 2, the authors should explicitly explain the fundamental findings observed in these ROC and Precision-Recall curves within the Results section.

Minor Revisions

Bibliography: The references do not currently follow the Vancouver format required by the journal.

Glossary: It would be appropriate to include a glossary or figure legend defining the metrics used (AUROC, AUPRC, F1-score) to assist clinical readers in the comprehension of the manuscript

Reviewer #2: 1. The authors used Integrated Gradients method for model explaination, but cohort-level feature importance based on absolute attributions can obscure directionality (protective vs risky). Please include individual examples showing feature importance over time and propose how explanations would be displayed to clinicians in real-time.

2. While AUROCs are good, but the AUPRCs, PPVs are not high, largely due to low event prevalence. The authors discuss alarm fatigue, but further clarification is needed on how clinicians should interpret risk outputs in practice. For example, what magnitude of predicted probability would justify clinical intervention? How many false alerts per hour would be acceptable operationally? Providing a clearer clinical integration pathway would improve translational clarity.

3. The manuscript frequently describes the proposed models as “lightweight,” but this claim would benefit from clearer quantitative justification. I recommend providing objective evidence such as parameter counts, memory footprint, inference latency, and computational requirements on representative edge hardware to substantiate the lightweight deployment claim.

4. The authors emphasize edge-device compatibility but lacks details on implementation challenges in actual transport environments. For example: How would missing data be handled in real time? What is the fallback mechanism if the model fails to generate a prediction?

Reviewer #3: The authors proposed two machine learning models to predict deterioration during pediatric critical care transport. The model has the potential to improve clinical care by earlier intervention. The methods are mostly robust, and the results seem promising. I appreciate the reporting of the results, which included metrics that are actionable in clinical settings and models explanations to help with the interpretability of the tools. However, I have some concerns.

Major Issues:

1. The manuscript lacked details on the model architecture. I was not clear on what each variant was tested until I read the supplements. The figures depicting the model architecture was confusing as well, especially around vital signs. For example, how was the 18x924 shape was obtained, and subsequently how does preprocessing lead to 36x924?

2. Connected to point 1, how were the vitals input processed for the transformer blocks? The methods section also lacked details on that.

3. While I appreciate that the authors are transparent with the amount of missing data and the small dataset size, the lack of data does show up in the results in terms of how jagged the curves are Figure 2. It was not clear how the confidence interval was calculated also.

4. The authors mentioned the operational constraint that only two recording devices are available and technical issues limited the amount of data available. Is that the case across health systems? As the models hedges on data being available, those sound like big hurdles to overcome even for model validation and deployments. I am also concerned with how such issues can create bias in the data and how well the results translate to a larger set of patients. The authors should elaborate and address these concerns in the discussion.

5. How much does vitals outside the 15 min window contribute? It would be interesting to see another variant using only the 15 min vitals.

6. As the models predict events, how would such predictions lead to actions? Discussion should be expanded with how clinicians could benefit from this tool.

Minor Issues:

1. Figures are in poor quality. The AUROC/PR curves are very pixelated. The overview figure should be condensed to only the key steps in the work.

2. There’s a reference error in Supplementary Table 1. I am seeing Error! Reference source not found under the Input Features column.

3. The bolding in Table 4 is confusing. The AUROC bolded is not the best model.

4. It is not clear what the feature set is, or what is being reduced in the reduced variants.

**********

6. PLOS authors have the option to publish the peer review history of their article (what does this mean?). If published, this will include your full peer review and any attached files.

**Do you want your identity to be public for this peer review?** If you choose “no”, your identity will remain anonymous but your review may still be made public.

For information about this choice, including consent withdrawal, please see our Privacy Policy.

Reviewer #1: **Yes:** Maria del Pilar Arias.MD. PICU staff Hospital de Niños Ricardo Gutierrez. Asesor Committee of Management , Quality and Data. Argentine Society of Intensive Care

Reviewer #2: No

Reviewer #3: No

**********

[NOTE: If reviewer comments were submitted as an attachment file, they will be attached to this email and accessible via the submission site. Please log into your account, locate the manuscript record, and check for the action link "View Attachments". If this link does not appear, there are no attachment files.]

**Figure resubmission:**

While revising your submission, we strongly recommend that you use PLOS’s NAAS tool (https://ngplosjournals.pagemajik.ai/artanalysis) to test your figure files. NAAS can convert your figure files to the TIFF file type and meet basic requirements (such as print size, resolution), or provide you with a report on issues that do not meet our requirements and that NAAS cannot fix.

After uploading your figures to PLOS’s NAAS tool - https://ngplosjournals.pagemajik.ai/artanalysis, NAAS will process the files provided and display the results in the "Uploaded Files" section of the page as the processing is complete. If the uploaded figures meet our requirements (or NAAS is able to fix the files to meet our requirements), the figure will be marked as "fixed" above. If NAAS is unable to fix the files, a red "failed" label will appear above. When NAAS has confirmed that the figure files meet our requirements, please download the file via the download option, and include these NAAS processed figure files when submitting your revised manuscript.

**Reproducibility:**

To enhance the reproducibility of your results, we recommend that authors of applicable studies deposit laboratory protocols in protocols.io, where a protocol can be assigned its own identifier (DOI) such that it can be cited independently in the future. Additionally, PLOS ONE offers an option to publish peer-reviewed clinical study protocols. Read more information on sharing protocols at https://plos.org/protocols?utm_medium=editorial-email&utm_source=authorletters&utm_campaign=protocols

# Author comment: Author response to Decision Letter 1

**DOI:** 10.1371/journal.pdig.0001410.r002

17 Feb 2026

Attachment Submitted filename: Response to Reviewers v0.1.docx

# Peer review: Decision Letter 1

**DOI:** 10.1371/journal.pdig.0001410.r003

**Authors:** Chenxi Yang

19 Mar 2026

PDIG-D-25-01001R1

Real-time prediction of cardiorespiratory deterioration during paediatric critical care transport using interpretable machine learning

PLOS Digital Health

Dear Dr. Kapur,

Thank you for submitting your manuscript to PLOS Digital Health. After careful consideration, we feel that it has merit but does not fully meet PLOS Digital Health's publication criteria as it currently stands. Therefore, we invite you to submit a revised version of the manuscript that addresses the points raised during the review process.

Please submit your revised manuscript by Apr 18 2026 11:59PM. If you will need more time than this to complete your revisions, please reply to this message or contact the journal office at digitalhealth@plos.org. When you're ready to submit your revision, log on to https://www.editorialmanager.com/pdig/ and select the 'Submissions Needing Revision' folder to locate your manuscript file.

Please include the following items when submitting your revised manuscript:

* A letter that responds to each point raised by the editor and reviewer(s). You should upload this letter as a separate file labeled 'Response to Reviewers'. This file does not need to include responses to any formatting updates and technical items listed in the 'Journal Requirements' section below.

* A marked-up copy of your manuscript that highlights changes made to the original version. You should upload this as a separate file labeled 'Revised Manuscript with Track Changes'.

* An unmarked version of your revised paper without tracked changes. You should upload this as a separate file labeled 'Manuscript'.

If you would like to make changes to your financial disclosure, competing interests statement, or data availability statement, please make these updates within the submission form at the time of resubmission. Guidelines for resubmitting your figure files are available below the reviewer comments at the end of this letter.

We look forward to receiving your revised manuscript.

Kind regards,

Chenxi Yang, Ph.D.

Academic Editor

PLOS Digital Health

Chenxi Yang

Academic Editor

PLOS Digital Health

Leo Anthony Celi

Editor-in-Chief

PLOS Digital Health

orcid.org/0000-0001-6712-6626

**Journal Requirements:**

If the reviewer comments include a recommendation to cite specific previously published works, please review and evaluate these publications to determine whether they are relevant and should be cited. There is no requirement to cite these works unless the editor has indicated otherwise.

**Additional Editor Comments (if provided):**

[Note: HTML markup is below. Please do not edit.]

**Reviewers' Comments:**

Reviewer's Responses to Questions

**Comments to the Author**

1. If the authors have adequately addressed your comments raised in a previous round of review and you feel that this manuscript is now acceptable for publication, you may indicate that here to bypass the “Comments to the Author” section, enter your conflict of interest statement in the “Confidential to Editor” section, and submit your "Accept" recommendation.

Reviewer #1: All comments have been addressed

Reviewer #2: All comments have been addressed

Reviewer #3: All comments have been addressed

**********

2. Does this manuscript meet PLOS Digital Health’s publication criteria? Is the manuscript technically sound, and do the data support the conclusions? The manuscript must describe methodologically and ethically rigorous research with conclusions that are appropriately drawn based on the data presented.

Reviewer #1: Yes

Reviewer #2: Yes

Reviewer #3: Partly

**********

3. Has the statistical analysis been performed appropriately and rigorously?

Reviewer #1: Yes

Reviewer #2: Yes

Reviewer #3: Yes

**********

4. Have the authors made all data underlying the findings in their manuscript fully available (please refer to the Data Availability Statement at the start of the manuscript PDF file)?

The PLOS Data policy requires authors to make all data underlying the findings described in their manuscript fully available without restriction, with rare exception. The data should be provided as part of the manuscript or its supporting information, or deposited to a public repository. For example, in addition to summary statistics, the data points behind means, medians and variance measures should be available. If there are restrictions on publicly sharing data—e.g. participant privacy or use of data from a third party—those must be specified.

Reviewer #1: Yes

Reviewer #2: Yes

Reviewer #3: No

**********

5. Is the manuscript presented in an intelligible fashion and written in standard English?

PLOS Digital Health does not copyedit accepted manuscripts, so the language in submitted articles must be clear, correct, and unambiguous. Any typographical or grammatical errors should be corrected at revision, so please note any specific errors here.

Reviewer #1: Yes

Reviewer #2: Yes

Reviewer #3: Yes

**********

6. Review Comments to the Author

Please use the space provided to explain your answers to the questions above. You may also include additional comments for the author, including concerns about dual publication, research ethics, or publication ethics. (Please upload your review as an attachment if it exceeds 20,000 characters)

Reviewer #1: Dear Authors,

Thank you for the detailed revision of your manuscript, "Real-time prediction of cardiorespiratory deterioration during paediatric critical care transport using interpretable machine learning" (PDIG-D-25-01001R1).

The effort to restructure the document and improve the methodological clarity is well-received. However, there are a few points that still require your attention before the manuscript can be finalized:

1. Evaluation of Bias and Fairness: regarding the handling of demographic variables and potential bias: it is important to emphasize that excluding sensitive variables is not necessarily the most effective way to mitigate bias. Machine learning models frequently learn from implicit biases already embedded in clinical data (such as practice patterns or systemic inequities). Therefore, a more robust way to evaluate and address bias is to explicitly analyze and report the model's performance across different subgroups, for example patients with missing race-etnicity. This ensures that the predictive accuracy remains consistent regardless of the patient's demographic background, rather than assuming that removing the variable eliminates the underlying bias.

Minor comments

2. Figure References: in the "Data Sources" section, specifically at line 8, you mention a visual representation of the data, but the specific figure number is missing. Please ensure this reference is completed to maintain the manuscript's technical clarity.

3. Minor Editorial Note: I noticed that specific tables are still being cited within the Discussion section. The Discussion should focus on the conceptual interpretation and clinical implications of your findings. I suggest avoiding direct citations of tables in this section to allow for a more fluid narrative, as the primary data has already been presented and referenced in the Results section.

I look forward to seeing these final adjustments.

Best regards,

Reviewer #2: I have no further comments.

Reviewer #3: (No Response)

**********

7. PLOS authors have the option to publish the peer review history of their article (what does this mean?). If published, this will include your full peer review and any attached files.

**Do you want your identity to be public for this peer review?** If you choose “no”, your identity will remain anonymous but your review may still be made public.

For information about this choice, including consent withdrawal, please see our Privacy Policy.

Reviewer #1: **Yes:** Maria del Pilar Arias

Reviewer #2: No

Reviewer #3: No

**********

[NOTE: If reviewer comments were submitted as an attachment file, they will be attached to this email and accessible via the submission site. Please log into your account, locate the manuscript record, and check for the action link "View Attachments". If this link does not appear, there are no attachment files.]

**Figure resubmission:**

While revising your submission, we strongly recommend that you use PLOS’s NAAS tool (https://ngplosjournals.pagemajik.ai/artanalysis) to test your figure files. NAAS can convert your figure files to the TIFF file type and meet basic requirements (such as print size, resolution), or provide you with a report on issues that do not meet our requirements and that NAAS cannot fix.

After uploading your figures to PLOS’s NAAS tool - https://ngplosjournals.pagemajik.ai/artanalysis, NAAS will process the files provided and display the results in the "Uploaded Files" section of the page as the processing is complete. If the uploaded figures meet our requirements (or NAAS is able to fix the files to meet our requirements), the figure will be marked as "fixed" above. If NAAS is unable to fix the files, a red "failed" label will appear above. When NAAS has confirmed that the figure files meet our requirements, please download the file via the download option, and include these NAAS processed figure files when submitting your revised manuscript.

**Reproducibility:**

To enhance the reproducibility of your results, we recommend that authors of applicable studies deposit laboratory protocols in protocols.io, where a protocol can be assigned its own identifier (DOI) such that it can be cited independently in the future. Additionally, PLOS ONE offers an option to publish peer-reviewed clinical study protocols. Read more information on sharing protocols at https://plos.org/protocols?utm_medium=editorial-email&utm_source=authorletters&utm_campaign=protocols

# Author comment: Author response to Decision Letter 2

**DOI:** 10.1371/journal.pdig.0001410.r004

2 Apr 2026

Attachment Submitted filename: Response to Reviewers v0.2.docx

# Peer review: Decision Letter 2

**DOI:** 10.1371/journal.pdig.0001410.r005

**Authors:** Chenxi Yang

19 Apr 2026

Real-time prediction of cardiorespiratory deterioration during paediatric critical care transport using interpretable machine learning

PDIG-D-25-01001R2

Dear Dr Kapur,

We are pleased to inform you that your manuscript 'Real-time prediction of cardiorespiratory deterioration during paediatric critical care transport using interpretable machine learning' has been provisionally accepted for publication in PLOS Digital Health.

Before your manuscript can be formally accepted you will need to complete some formatting changes, which you will receive in a follow-up email from a member of our team.

Please note that your manuscript will not be scheduled for publication until you have made the required changes, so a swift response is appreciated.

IMPORTANT: The editorial review process is now complete. PLOS will only permit corrections to spelling, formatting or significant scientific errors from this point onwards. Requests for major changes, or any which affect the scientific understanding of your work, will cause delays to the publication date of your manuscript.

If your institution or institutions have a press office, please notify them about your upcoming paper to help maximize its impact. If they'll be preparing press materials, please inform our press team as soon as possible -- no later than 48 hours after receiving the formal acceptance. Your manuscript will remain under strict press embargo until 2 pm Eastern Time on the date of publication. For more information, please contact digitalhealth@plos.org.

Thank you again for supporting Open Access publishing; we are looking forward to publishing your work in PLOS Digital Health.

Best regards,

Chenxi Yang, Ph.D.

Academic Editor

PLOS Digital Health

***********************************************************

**Additional Editor Comments (if provided):**

The authors have addressed the concerns from all reviewers.

**Reviewer Comments (if any, and for reference):**

Reviewer's Responses to Questions

**Comments to the Author**

1. If the authors have adequately addressed your comments raised in a previous round of review and you feel that this manuscript is now acceptable for publication, you may indicate that here to bypass the “Comments to the Author” section, enter your conflict of interest statement in the “Confidential to Editor” section, and submit your "Accept" recommendation.

Reviewer #1: All comments have been addressed

**********

2. Does this manuscript meet PLOS Digital Health’s publication criteria? Is the manuscript technically sound, and do the data support the conclusions? The manuscript must describe methodologically and ethically rigorous research with conclusions that are appropriately drawn based on the data presented.

Reviewer #1: Yes

**********

3. Has the statistical analysis been performed appropriately and rigorously?

Reviewer #1: Yes

**********

4. Have the authors made all data underlying the findings in their manuscript fully available (please refer to the Data Availability Statement at the start of the manuscript PDF file)?

The PLOS Data policy requires authors to make all data underlying the findings described in their manuscript fully available without restriction, with rare exception. The data should be provided as part of the manuscript or its supporting information, or deposited to a public repository. For example, in addition to summary statistics, the data points behind means, medians and variance measures should be available. If there are restrictions on publicly sharing data—e.g. participant privacy or use of data from a third party—those must be specified.

Reviewer #1: Yes

**********

5. Is the manuscript presented in an intelligible fashion and written in standard English?

PLOS Digital Health does not copyedit accepted manuscripts, so the language in submitted articles must be clear, correct, and unambiguous. Any typographical or grammatical errors should be corrected at revision, so please note any specific errors here.

Reviewer #1: Yes

**********

6. Review Comments to the Author

Please use the space provided to explain your answers to the questions above. You may also include additional comments for the author, including concerns about dual publication, research ethics, or publication ethics. (Please upload your review as an attachment if it exceeds 20,000 characters)

Reviewer #1: Thank you for the detailed responses and the revisions made to the manuscript. All my previous concerns have been addressed satisfactorily. The study provides valuable insights into real-time prediction during pediatric transport, and I believe it is now ready for publication.

**********

7. PLOS authors have the option to publish the peer review history of their article (what does this mean?). If published, this will include your full peer review and any attached files.

**Do you want your identity to be public for this peer review?** If you choose “no”, your identity will remain anonymous but your review may still be made public.

For information about this choice, including consent withdrawal, please see our Privacy Policy.

Reviewer #1: **Yes:** Maria del Pilar Arias Lo`pez

**********
